# A direct comparison of interphase FISH versus low-coverage single cell sequencing to detect aneuploidy reveals respective strengths and weaknesses

**DOI:** 10.1038/s41598-019-46606-w

**Published:** 2019-07-19

**Authors:** Grasiella A. Andriani, Elaine Maggi, Daniel Piqué, Samuel E. Zimmerman, Moonsook Lee, Wilber Quispe-Tintaya, Alexander Maslov, Judith Campisi, Jan Vijg, Jessica C. Mar, Cristina Montagna

**Affiliations:** 1 Department of Genetics, Albert Einstein College of Medicine, Bronx, NY 10461 USA; 2 Rutgers Cancer Institute of New Jersey, New Brunswick, NJ 08901 USA; 3 Department of Epidemiology and Population Health, Albert Einstein College of Medicine, Bronx, NY 10461 USA; 4 Australian Institute for Bioengineering and Nanotechnology, the University of Queensland, Brisbane, QLD 4072 Australia; 5 Buck Institute for Research on Aging, 8001 Redwood Boulevard, Novato, California USA; 6 Biosciences Division, Lawrence Berkeley National Laboratory, 1 Cyclotron Road, Berkeley, California USA; 7 Department of Pathology, Albert Einstein College of Medicine, Bronx, NY 10461 USA

**Keywords:** Genomic analysis, Cytogenetics

## Abstract

Aneuploidy has been reported to occur at remarkably high levels in normal somatic tissues using Fluorescence *In Situ* Hybridization (FISH). Recently, these reports were contradicted by single-cell low-coverage whole genome sequencing (scL-WGS) analyses, which showed aneuploidy frequencies at least an order of magnitude lower. To explain these seemingly contradictory findings, we used both techniques to analyze artificially generated mock aneuploid cells and cells with natural random aneuploidy. Our data indicate that while FISH tended to over-report aneuploidies, a modified 2-probe approach can accurately detect low levels of aneuploidy. Further, scL-WGS tends to underestimate aneuploidy levels, especially in a polyploid background.

## Introduction

Aneuploidy, an abnormal chromosome number, is commonly deleterious and observed in cancer and certain congenital disorders. A high frequency of aneuploidy has also been reported in the developing and adult brain (~3–35%)^1–6^ and the liver (~25–70%)^7–10^, where it is hypothesized to provide a selective advantage by generating genetic diversity. We previously reported increased aneuploidy in the aging mouse brain and proposed that it contributed to age-related neurodegeneration^3^.

The aforementioned studies applied molecular cytogenetic techniques (i.e., Fluorescence *In Situ* Hybridization–FISH, and Spectral Karyotyping–SKY) to measure aneuploidy. More recently, studies using single-cell low-coverage whole genome sequencing (scL-WGS) to analyze similar tissues reported much lower aneuploidy levels (0.7–2.2% in the adult human and murine brain and <5% in murine hepatocytes)^11–13^. These studies suggested that somatic aneuploidy is much less common than previously reported, and that conventional FISH-based methods were prone to artifacts vastly overestimating somatic aneuploidy in these tissues^14^. To elucidate the source of these discrepancies, we directly compared the two approaches to assess strengths and weaknesses of both techniques. We performed interphase FISH (iFISH) and scL-WGS on cells derived from parallel pools of samples of controlled mock aneuploidies and a model of random aneuploidy. FISH, when used with one probe/chromosome significantly over estimate the frequency of aneuploid cells, but a dual probe per chromosome configuration allows for a sensitive and reproducible assessment of chromosome numerical imbalances, albeit limited to the few chromosomes tested in each hybridization. scL-WGS consent copy number analysis on all the chromosome complement in the same cell, but significantly underestimated aneuploidy in a polypoid background (complex aneuploidy).

## Results and Discussion

Many powerful molecular cytogenetic techniques are available with different levels of resolution and throughput to study genome rearrangements and copy number changes. Some are specific for metaphase chromosome analysis (i.e. G-banding, Spectral Karyotyping, multiplex-FISH, multicolor banding); others are suitable to analyze interphase or non-diving cells (i.e. comparative genomic hybridization and iFISH)^15^. As many different types of samples can be analyzed (i.e. cells in suspension, tissue sections, core biopsies), iFISH is perhaps the most versatile. iFISH is amenable to high throughput analyses and probes for regions of interest can be easily generated to customize the assay. Indeed, iFISH has been applied to study different attributes of the genome (i.e. pericentromeric regions, identification of euchromatic genomic loci, telomere analysis, chromosome painting, and simultaneous visualization of the complete set of chromosomes)^16^. A straightforward approach is to utilize iFISH with locus specific probes for chromosome enumeration which makes it possible to detect numerical chromosome imbalances (aneuploidy and polyploidy) in vast cell populations. iFISH, like other FISH based assays, bear limitations such as high backgrounds, localized lack of hybridization signals, variation in hybridization efficiency and probe clustering that can lead to false positive or false negative results^17,18^.

To overcome these known FISH limitations, we designed and validated an interphase FISH (iFISH) assay that used two probes, labeled by different fluorophores, for each of two chromosomes tested^3,5,19^ (Supplementary Table 1). Cells were scored for probe signals at both loci, and were classified as aneuploid only when gains or losses were detected at both probes, greatly reducing the number of false positives. Indeed, we confirmed that the use of only one probe/chromosome is significantly more prone to false positives (2.3% +/−0.7% detected in the mouse adult brain when using one probe, versus 0.9% +/−0.6% when using the 2-probe iFISH, *p* = 0.0073; – Supplementary Table S2), as suggested^3^.

Using the same 2-probe/chromosome iFISH assay to assess aneuploidy, we found high levels in the cortex of old mice (up to 8.4% +/− 1.1% for chromosome 18). This level is unlikely to be an artifact because extremely low levels of aneuploidy (<0.7%) were observed in the cerebellum of the same mice^3,5^, supporting similar findings in the human brain^20^. Thus the 2-probe/chromosome iFISH assay is more accurate than the generally used molecular cytogenetic approaches, and is capable of detecting cell type- and brain region-specific differences in the level of aneuploidy.

The 2-probe FISH assay analyzes only two chromosomes simultaneously, necessitating extrapolation from only two chromosomes to the entire chromosome complement thereby reducing the potential for a comprehensive aneuploidy estimation. In order to analyze the entire chromosome complement, we directly compared our 2-probe iFISH assay with scL-WGS. For this analysis, we used primary fibroblasts that are trisomic for chromosomes 13 (T13), 18 (T18) or 21 (T21). All T13 (N = 9) and T21 (N = 9) cells were correctly identified as containing one additional copy of chromosomes 13 or 21 (Fig. S1). In T18 cells, scL-WGS detected 2 out of 9 cells (~22%) as diploid (Fig. S2). Indeed, when analyzed by FISH using a chromosome 18 probe, only 63% of the cells were scored as trisomic for chromosome 18, whereas 26.3% scored diploid and 5.3% scored tetrasomic 18. Thus, overall, the FISH results corroborated the scL-WGS findings for the detection of known single chromosome aneuploidy. Apart from chromosome 18 trisomy, both methods detected no aneuploidies across other chromosomes.

We next asked whether the low levels of aneuploidy repeatedly reported in the brain and liver using scL-WGS could be due to underestimation, such as through saturation of DNA yield during single-cell whole genome amplification (WGA). To test the sensitivity of scL-WGS under different conditions, we devised a precisely controlled approach to detect polyploidy and complex aneuploidy (herein referred to as aneuploidy in a polyploid background). This approach is particularly useful because aneuploidy is found in a highly polyploid background in both human^8^ and mouse^10^ hepatocytes, as well as the brain under physiological and pathological conditions^21,22^, albeit at lower frequency.

**Figure 1:**
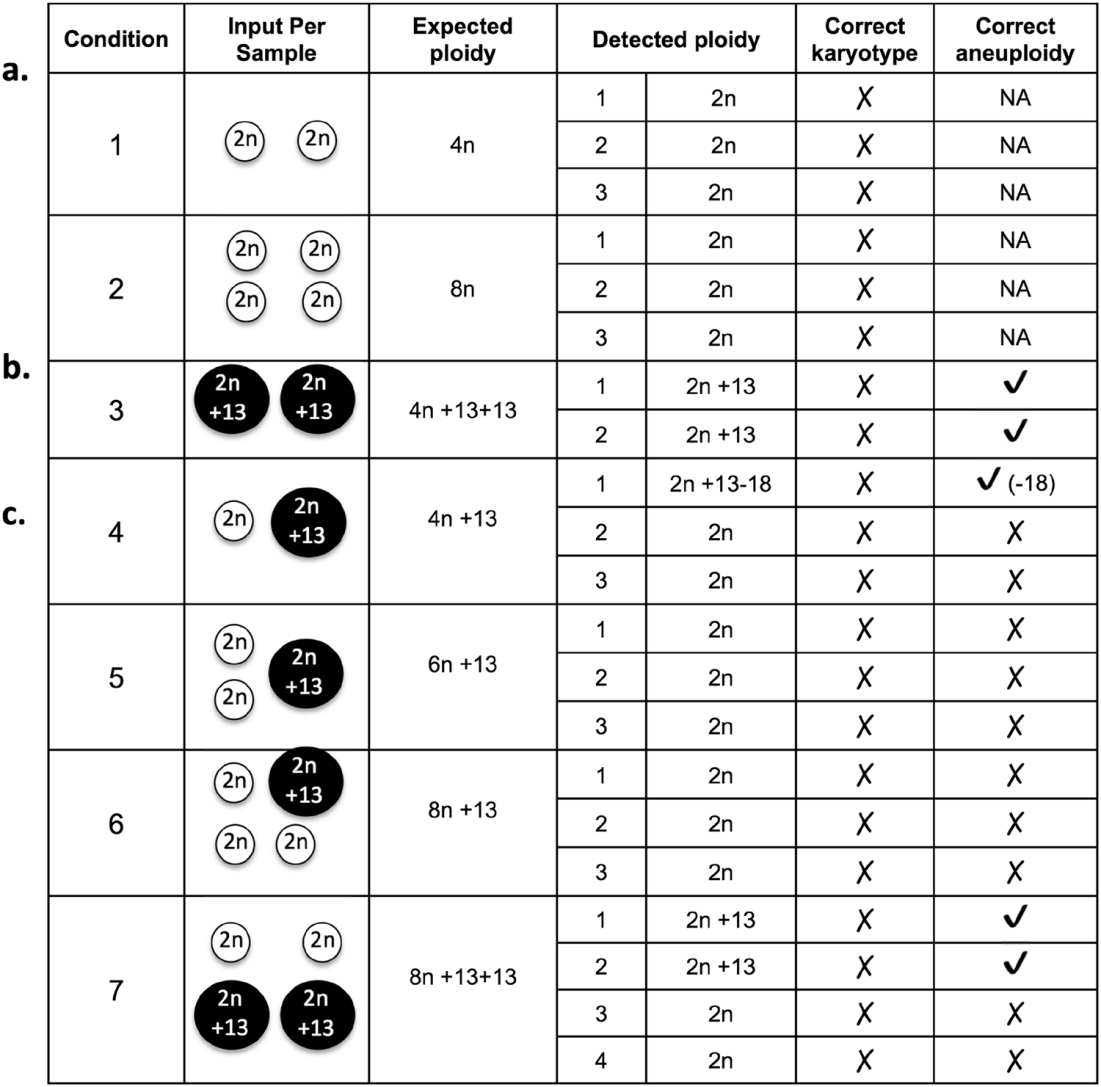
Mock aneuploidy and aneuploidy detected by scL-WGS. **(a)** Polyploidy, generated by combining two or four 2n cells into a tube prior to WGA. **(b,c)** Aneuploidy in a polyploid background, generated by combining 2n and T13 cells, as illustrated. The detected ploidy and aneuploidy are indicated to the right where detection of the correct karyotype and/or aneuploidy is also shown.

We created controlled mock aneuploid cells by isolating single cells with different known ploidy (diploid proliferating–PRO–cells and T13 cells) and combining them in the same tube, prior to WGA. This manipulation created experimental conditions that mimicked polyploidy (4n and 8n) and/or complex aneuploidy (Fig. 1).

scL-WGS failed to identify the correct chromosome complement for all of the mock aneuploidies. First, scL-WGS failed to distinguish between 4n and 8n, both of which were detected as 2n (Fig. 1a). Second, in the mock ploidy 4n + 13 + 13 (two T13 cells), scL-WGS failed to detect both of the extra copies of chromosome 13 but instead identified all cells as 2n + 13 (Figs 1b and S3). Third, and most importantly, in the other complex aneuploidy conditions, (i.e. chromosome 13 gain in 4n, 6n or 8n ploidy backgrounds), scL-WGS failed to detect either the correct ploidy and/or the correct copy number for chromosome 13 (Fig. 1c). Thus, for ploidy changes, scL-WGS sensitivity was 0%, albeit without false positive calls. For complex aneuploidy, scL-WGS sensitivity was 33.3% (5 of 15 cells were detected with the correct aneuploid chromosome) and 93.4% specific (we detected one cell with chromosome 18 loss). While loss of chromosome 18 might be a true event due to the cell’s attempt to maintain homeostasis, this cannot be confirmed, which highlights an additional limitation of scL-WGS. scL-WGS requires complete cell lysis, and, unlike FISH, constrains downstream technical validation (i.e. stripping and re-probing for additional chromosomes, capture from the slide followed by genomic analysis).

Adjusting the analytical pipeline for bin size, fixed versus variable bins and normalization method yielded similar results (Supplementary Table S3). Likewise, analysis of mock aneuploid cells using additional bioinformatics tools (Aneufinder or Ion Reporter software) returned similar results, emphasizing the low sensitivity of scL-WGS for detecting polyploidy^11^ and complex aneuploidies (Figs S4, S5). We attempted to computationally resolve the scL-WGS data to deliver the expected ploidy by: (i) checking for differences in mean read number across cells known to have different ploidy states; (ii) calling chromosomal ploidy using an outlier detection approach that normalizes across read counts and used the young cells which are mainly diploid as a reference (Fig. S6). Both attempts failed to detect the expected ploidies.

**Figure 2:**
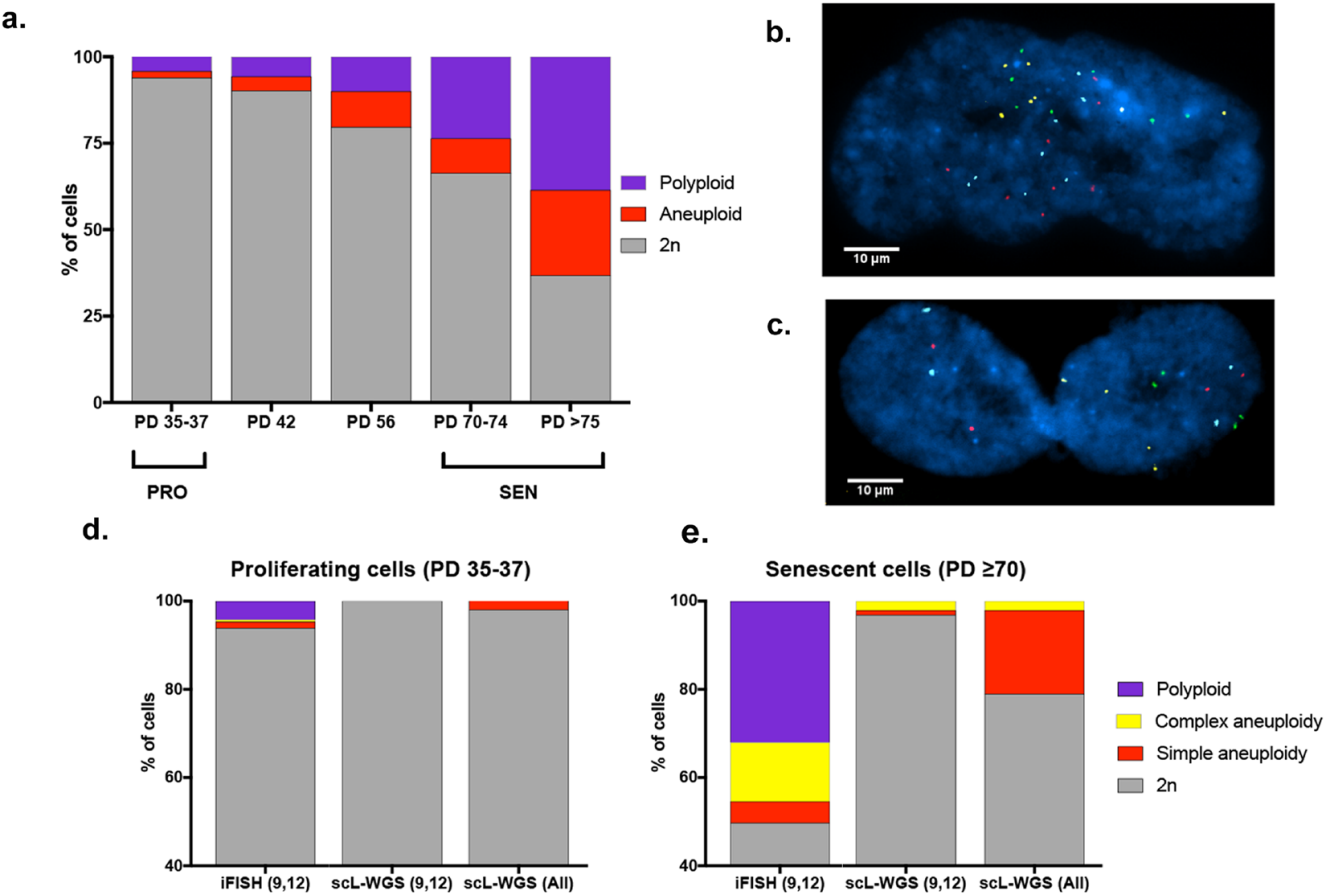
Ploidy analysis of IMR-90 human primary fibroblasts by iFISH and scL-WGS. (**a)** Ploidy distribution at different population doublings (PDs) of IMR-90 cells, measured by 4-color iFISH. Proliferating cells (PRO, N = 605) were analyzed at population doubling levels (PDL) 35–37 and replicatively senescent cells (SEN, N = 905) analyzed from PDL 70 and beyond. **(b,c)** Representative images of a cell **(b)** with complex aneuploidy and **(c)** binucleated tetraploid cell. **(d)** Percentage of non-diploid (Not 2n) cells detected by iFISH and scL-WGS in PRO (N = 51) and **(e)** SEN (N = 95) cells. scL-WGS (All) refers to the levels of Not 2n cells considering all chromosomes. scL-WGS (9,12) refers to the levels of Not 2n cells considering only chromosomes 9 and 12, which were also analyzed by iFISH.

To further test the ability of scL-WGS to detect complex aneuploidies relative to iFISH, we analyzed replicatively senescent (SEN) normal human fibroblasts (IMR-90), which accumulate ploidy changes as they approach senescence due to repeated cell division in culture^23^. 4-color iFISH using probes for chromosomes 9 and 12 detected ~6% non-diploid (Not 2n cells) cells in proliferating low passage (35–37 population doublings) cells (PRO). In the senescent (SEN) cultures, we detected 31–63% Not 2n cells (Fig. 2a). Within the Not 2n population, the percentage of polyploid cells was 21–38% and that of aneuploid cells was 9–26%. The frequency of polyploidy detected by iFISH in IMR-90 cells during repeated cell division is concordant with previously reported frequencies of primary fibroblasts in culture^24–28^. The extent of ploidy changes varied greatly among cells, from 1 to 17 copies of a chromosome (Supplementary Table S4). Moreover, ~27% of (SEN) cells were binucleated (Fig. 2b,c), which mimics the state of many hepatocytes *in vivo*^9,10^.

When scL-WGS was used to analyze IMR-90 (PRO) cells, we detected only 1 cell (of 51 cells analyzed) with a gain of 2 copies of chromosome 22 (1.9% Not 2n cells) (Figs 2d and S7). No ploidy changes were detected for chromosomes 9 or 12 by scL-WGS (0%), in contrast to the iFISH results using chromosomes 9 or 12 specific probes (~6%) (*p* = 0.0231) as well as contrary to ploidy changes known to occur in primary fibroblasts in culture^24–28^. Accordingly, scL-WGS analysis of (SEN) cells detected a significantly (p < 0.0001) lower percentage of Not 2n cells (~21%) than that detected by iFISH (average 47%) (Figs 2e and S8). When only cells aneuploid for chromosomes 9 and 12 were counted, the levels of Not 2n cells detected by scL-WGS in IMR90(SEN) cells was ~3.2% (3 of 95 cells). Only 2 cells (~2%) were detected as complex aneuploidy, and no polyploid cells were found. These results conflict substantially with the results obtained by iFISH and previously reported findings^29^, in which complex aneuploidy was present at a frequency of >10% and polyploidy was detected at a frequency of >30%.

The scL-WGS data was collected by ultra-low coverage sequencing (0.01–0.05X) across the genome. While low, this density is within the depth of coverage (0.01–0.5X) assumed to provide chromosome copy number calls with confidence^30^. However, accurate copy number assessment is difficult when >99% of each genome lacks sequencing coverage; this experimental limitation could contribute to the observed lack of sensitivity. Indeed, in the mock aneuploidy condition (4n + 13 + 13) with successful detection of chromosome 13 gain by scL-WGS, the percentage of the genome corresponding to a +13 is ~1.8% of the total cell DNA (GRCh37/hg19), which is the same as in 2n + 13 cells. In mock conditions #4 (4n + 13) and #7 (8n + 13 + 13) the percentage of relative genome mapping to +13 drops to ~0.9%. At this frequency scL-WGS detects the correct aneuploidy in 43% of the cells. In all other mock aneuploid conditions the frequency of genome corresponding to +13 was lower (~0.6% in 6n + 13 cells and ~0.46% in 8n + 13 cells). Thus, when the percentage of genome mapping to the chromosome gain is found at ~1.8% frequency scL-WGS can detect the correct aneuploidy. The efficiency drops to ~50% when the frequency of the aneuploidy chromosome is ~0.9%, below which scL-WGS is unable to discriminate chromosome copy number changes.

Moreover, WGA likely contributes to decreased scL-WGS sensitivity in a polyploid background. We routinely observe significantly higher DNA yield when amplifying mock aneuploid or (SEN), relative to (PRO), cells **(**Fig. S9a). In addition, quantifying relative DNA amounts through WGA shows that (SEN) cells yield significantly different DNA amounts than (PRO) cells only in the first few amplification cycles (Fig. S9b), after which DNA content differences are lost. Thus, to avoid over amplification, polyploid cells require much lower amplification cycles than those commonly recommended by WGA protocols (e.g., the PicoPLEX assay recommends 14 amplification cycles) (Fig. S9b).

## Conclusions

Our results confirm that FISH based on only one probe does indeed overestimate aneuploidy levels. scL-WGS, on the other hand, appears incapable of detecting aneuploidy against a polyploid background, something that 2-probe iFISH can easily do. This limitation is likely due to the ultra-low sequencing depth, combined with the saturation of DNA content as result of over amplification. This limitation is particularly misleading for analyses of aneuploidy in the liver, where 50–80% of hepatocytes are polyploid^8^, and likely explains the significantly lower aneuploidy levels detected by scL-WGS^11^.

Since scL-WGS has enhanced capabilities relative to iFISH – that is, the possibility of analyzing all chromosomes at once rather than only a few–it should be the method of choice. Conceivably, further improvements in amplification protocols could reduce the lack of sensitivity we uncovered. For now, it is important to use a combination of methods in cases where complex karyotypes are likely present.

## Methods

### Aim, design and setting of the study

The goal of this study was to directly compare Fluorescence *In Situ* Hybridization (FISH) and single-cell low-coverage whole genome sequencing (scL-WGS) to identify strengths and weaknesses of both techniques in measuring whole chromosome aneuploidy in single cells. Parallel cultures of artificially generated mock aneuploid cells and cells with random aneuploidy (replicatively senescent (SEN) normal human fibroblasts) were analyzed by both technologies and the results were directly compared.

### Comparison between iFISH analysis using 1 probe/chromosome vs. 2 probes/chromosome

Cerebral cortex nuclei were isolated from adult mice and hybridized with probes for chromosome 1, 7 and 18, as previously described^3^. The percentages of Not 2n cells obtained with 1 or 2 probes/chromosome were compared for statistical significance using t-test.

### Human primary fibroblast (HPF) cultures and generation of SEN fibroblasts

IMR-90 cells were purchased from ATCC (CCL-186) at Population Doubling (PD) 25 (~passage 12) and cultured as described previously^23^. T13 (AG10292), T18 (AG12614) and T21 (AG07096) human primary fibroblasts were obtained from the Coriell Cell Repositories and cultured according to supplier’s protocols for no longer than 48 h, before being processed by scL-WGS or FISH. SEN fibroblasts were obtained by sub-culturing the same IMR-90 cells until they failed to reach confluency even after 2 weeks of culturing (minimum 70 PDs), as described previously^31,32^. At least 4 independent SEN cultures were analyzed for both FISH and scL-WGS. Senescence was confirmed by SA-βgal staining, BrdU incorporation and/or SASP expression^23^.

### 4-color iFISH analysis

Ploidy analysis for human chromosomes 9 and 12 in interphase fibroblasts was performed as previously described^23,33^. Bacterial Artificial Chromosome (BAC) clones used in this study are listed in Supplementary Table S1; BAC DNA was isolated and labelled by nick translation as we previously described^19^. Specificity of the probes was verified using XX or XY methaphase chromosome preparations obtained from peripheral blood (human) or from spleenocytes (mouse) as we described^3^.

### Single cells amplification and sequencing

Single cells were picked into 2.5 μl of PBS using the CellRaft (Cell Microsystems) single cell picking system following manufacture instructions. For the mock aneuploidy cells multiple cells were picked into the same tube to ensure the correct number and type of cell. DNA amplification was performed using the Rubicon genomics PicoPLEX WGA Kit (Cat # R30050) per manufacture instructions with the adjustment of final amplification cycles. Purification was carried on using AMPure beads at a 0.9X concentration. Due to the possibility of sensitivity loss as result of over amplification we performed a test amplification with SYBR Green 1 (Invitrogen, cat # S7563). Five cells each of proliferating IMR90, senescent IMR90, and trisomy 18 cells were amplified with 0.125X SYBR green dye. Based on the SYBR Green 1 amplification curve we tested 7, 8, and 9 amplification cycles since this was the point before plateau and found the yields to be sufficient for further use. All three conditions were successful in detecting trisomies in our control cells; thus 8 cycles were used for all following experiments. Following amplification, 300 ng of DNA was used to create Ion Torrent libraries using the NEBNext Fast DNA Fragmentation & Library Prep Set for Ion Torrent (Cat # E6285L) with a few minor modifications. The adapter ligation was completed with 3 μl of NEXTflex® DNA Barcodes (cat #NOVA-401004) and the final amplification step was omitted. Libraries were purified using AMPure beads then 250 bp fragments were size selected with the Invitrogen E-gel size selection system. The libraries were sequenced at an average of 0.2X coverage on the Ion Proton and sequences and aligned to the hg19 human genome using the Torrent Suite 5.2.2 software. Raw sequencing files can be access through the Sequence Read Archive (SRA) (https://www.ncbi.nlm.nih.gov/sra) portal under project ID (SRP158797: PRJNA487805).

#### FISH data analysis

Images representing at least 200 nuclei were randomly acquired and saved as.tiff composite files for both PRO and SEN cells from 3 or more independent experiments. Images were visually inspected and FISH signals manually counted blindly for both chromosomes. Analyzed cells were classified as diploid (2n) or not-diploid (Not 2n). The Not 2n population was further divided into polyploid (cells containing the same number of copies for both chromosomes: >3n, 4n, 5n, 6n and higher) or aneuploid (cells containing any number of copies for each chromosome, as long as they do not coincide). A distinction can be made, within the aneuploid group, of cells that are aneuploid in a polyploid background (complex aneuploidy - i.e. any copy number combination that seem to result from a tetraploid intermediate state: 3 copies/4 copies, 4 copies/5 copies, 6 copies/8 copies and higher). Cells were classified accordingly the number of probe signals.

#### scL-WGS data analysis

BAM files generated from the Torrent Suite software were converted to BED files using the bedtools2 bamToBed function. The BED files were then uploaded into the aneuploidy identifier tool Ginkgo^30^. The analysis was performed using variable bins of 2.5 mb based on simulations of 150 bp reads with global segmentation. No additional parameters were changed. All cells with copy number changes in the number of at least one chromosome arm were counted as being aneuploidy. The results were also confirmed using different packages available for ultra-low coverage scL-WGS data, using the same parameters: Aneufinder^12^, and the Low-pass whole-genome aneuploidy w1.0 from the Ion Reporter Software 4.2. Workflow Version: 1.0.

### Computational analysis

A one-way ANOVA between experimental groups was used to test whether significant differences in the number of reads that map to mock ploidy cells existed. Statistical analyses were performed in R version 3.4.3. Code used to conduct these analyses is available upon request.

### Chromosomal binning and ploidy detection

To generate whole chromosome estimates of ploidy for each cell, a weighted average copy number (weighted by bin size) was applied to the inferred copy number output from Ginkgo for each chromosome. Next, diploid, early-passage fibroblasts were used to generate an “expected” proportion of reads that map to each human chromosome. The proportions for each chromosome were assumed to follow a Gaussian distribution. The “observed” proportion of reads that mapped to each chromosome within each mock cell were compared against the distribution of the “expected” proportion of reads for a given chromosome. We tested the possibility of extreme deviations on either side of this “expected” distribution and used a two-tailed z-score significance test (combined with the resulting p-value) to test for deviation in expected ploidy. Chromosomes in mock samples that had a significantly greater proportion of reads mapped to a chromosome (relative to “expected”) were called as a chromosomal gain, and chromosomes in mock samples that had a significantly fewer proportion of reads mapped to a chromosome were called as a chromosomal loss.

All cells were analyzed with different Ginkgo parameters: global (normalized read counts) versus independent normalization (sample with lowest IOD = the ratio between the read coverage variance and the mean) and fixed or variable bin size of 500Kb, 1 Mb, 2.5 Mb and 10 Mb for a total of 14 different Gingko settings tested.

For our analysis, we selected the 2.5 Mb variable window size with global normalization (setting #9). Gingko settings #1–5 performed slightly better in terms of correct aneuploidy calls, but they also called false positive chromosome gains or losses in the mock aneuploidy conditions or in the trisomic diploid cells. Ploidy number was assigned based on the lowest sum of squares error across continuous putative copy number state space. The near second best ploidy state was also incorrect in the majority of cases (with the exception being our chosen condition #9), in which the correct ploidy sum of squares was higher than the second best option.

#### qPCR on single cells

To further examine the differences in amplification of senescent cells vs proliferating cells we performed qPCR as listed above on 10 more proliferating and senescence single cells as well as five trisomy 21 cells. A student’s t-test was used to determine significant differences at each cycle between proliferating and senescent cells. One proliferating cell was not used in this analysis because it was determined to be an outlier by Grubb’s test (GraphPad Software).

All sequencing data are publicly available through the Sequence Read Archive (SRA^34^) under study ID: PRJNA487805 “scL-WGS of proliferative and senescent cells”.

## Supplementary information

Supplementary material
