# Supplementary material for: A direct comparison of interphase FISH versus low-coverage single cell sequencing to detect aneuploidy reveals respective strengths and weaknesses
Source: Sci Rep. 2019 Jul 19;9:10508. doi: 10.1038/s41598-019-46606-w (PMC6642082; doi:10.1038/s41598-019-46606-w)
Supplement: Supplementary file 1 — Supplementary material [file 41598_2019_46606_MOESM1_ESM.pdf]

A direct comparison of interphase FISH versus low-coverage single cell sequencing to detect aneuploidy reveals respective strengths and weaknesses

Grasiella A. Andriani[1*], Elaine Maggi[1,2*], Daniel Piqué[3], Samuel E. Zimmerman[3], Moonsook Lee[1], Wilber Quispe-Tintaya[1], Alexander Maslov[1], Judith Campisi[4,5], Jan Vijg[1], Jessica Mar[3], Cristina Montagna[1,6]

[1] Department of Genetics, Albert Einstein College of Medicine, Bronx, NY 10461, USA

[2] Rutgers Cancer Institute of New Jersey, New Brunswick, NJ 08901, USA

[3] Department of Systems biology, Albert Einstein College of Medicine, Bronx, NY 10461, USA

[4] Buck Institute for Research on Aging, 8001 Redwood Boulevard, Novato, California, USA

[5] Biosciences Division, Lawrence Berkeley National Laboratory, 1 Cyclotron Road, Berkeley, California USA

[6] Department of Pathology, Albert Einstein College of Medicine, Bronx, NY 10461, USA

*These two authors contributed equally to this work

Supplementary Figure S1

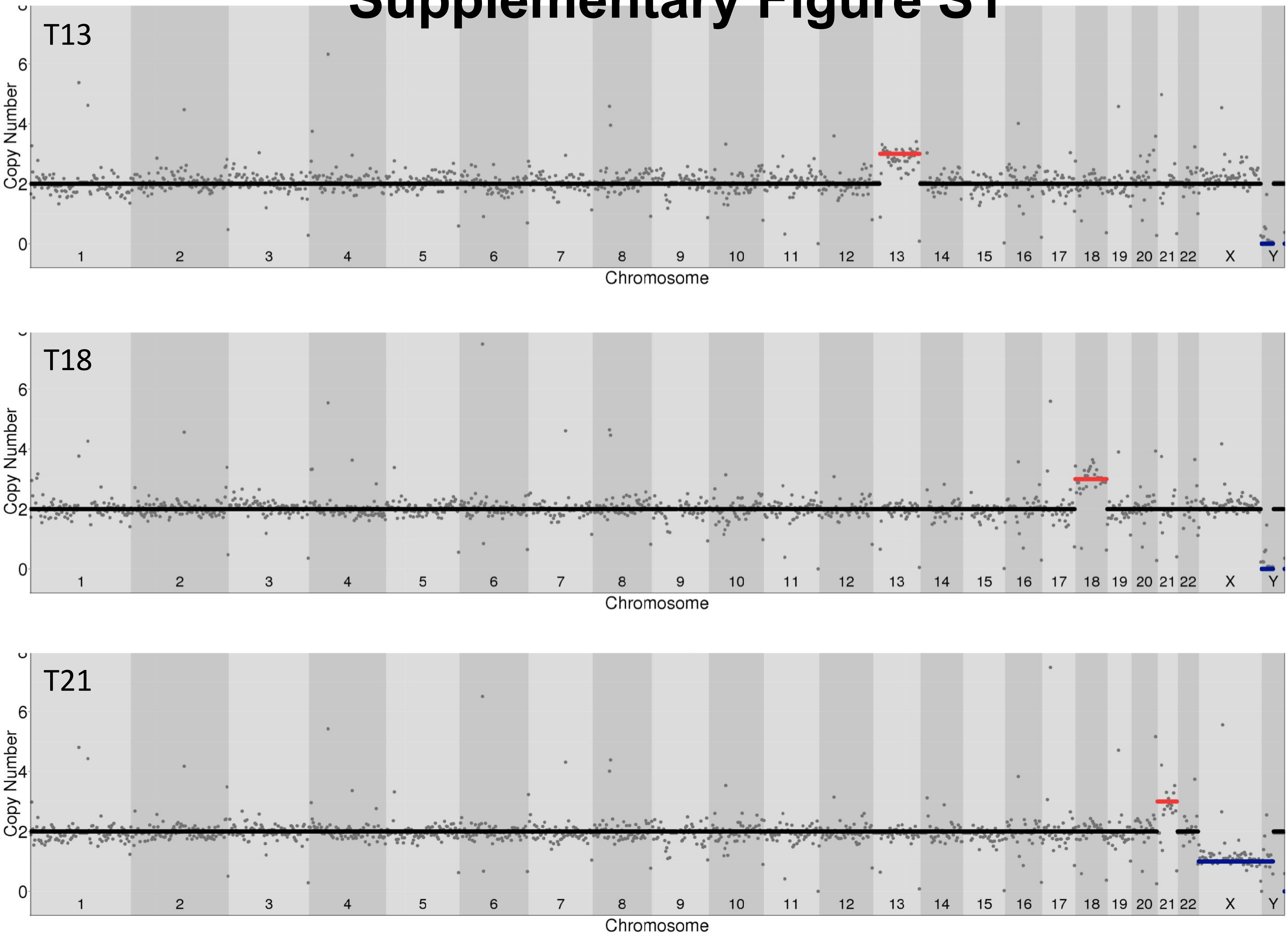

Supplementary Figure S2

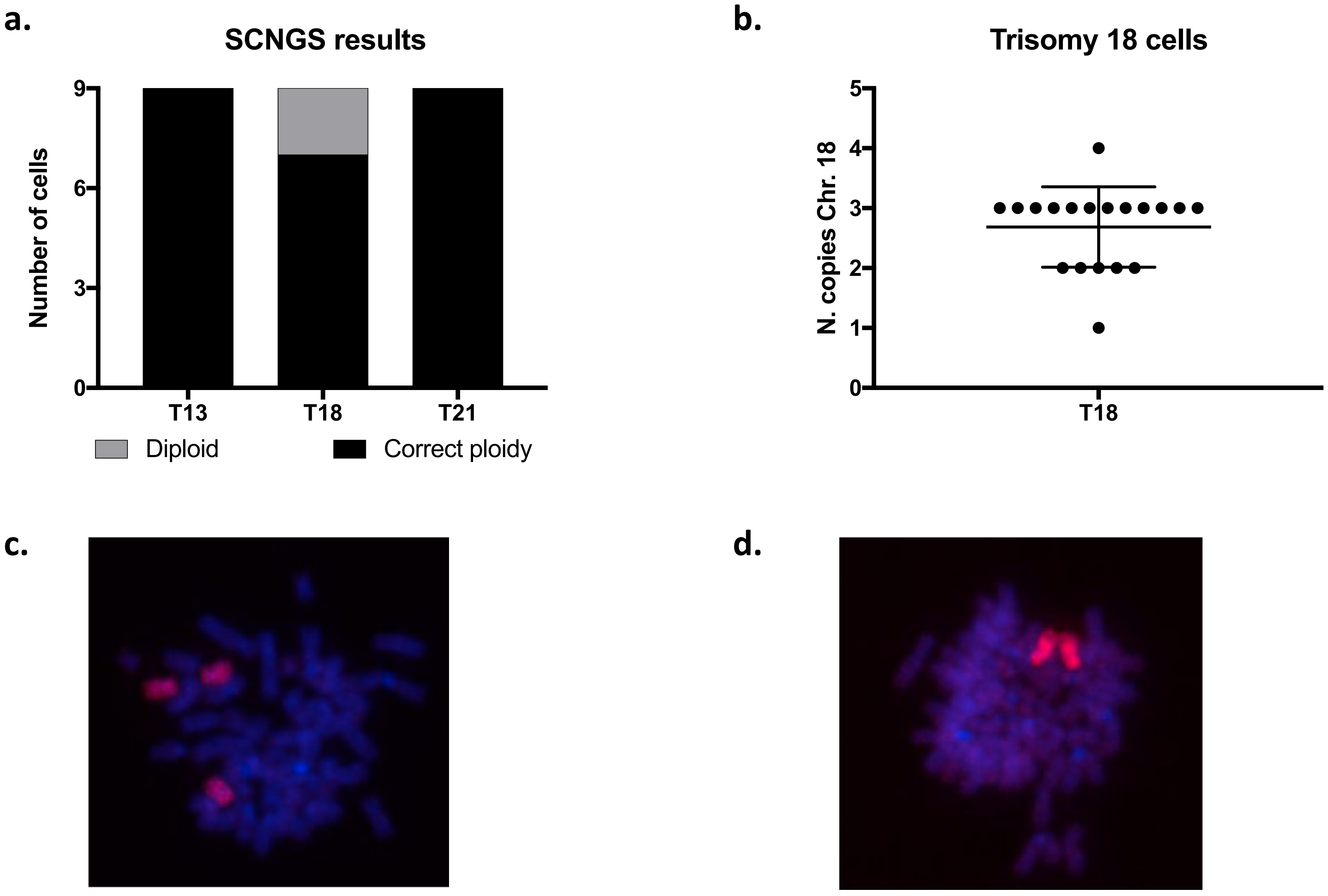

Supplementary Figure S3 (All mock cells)

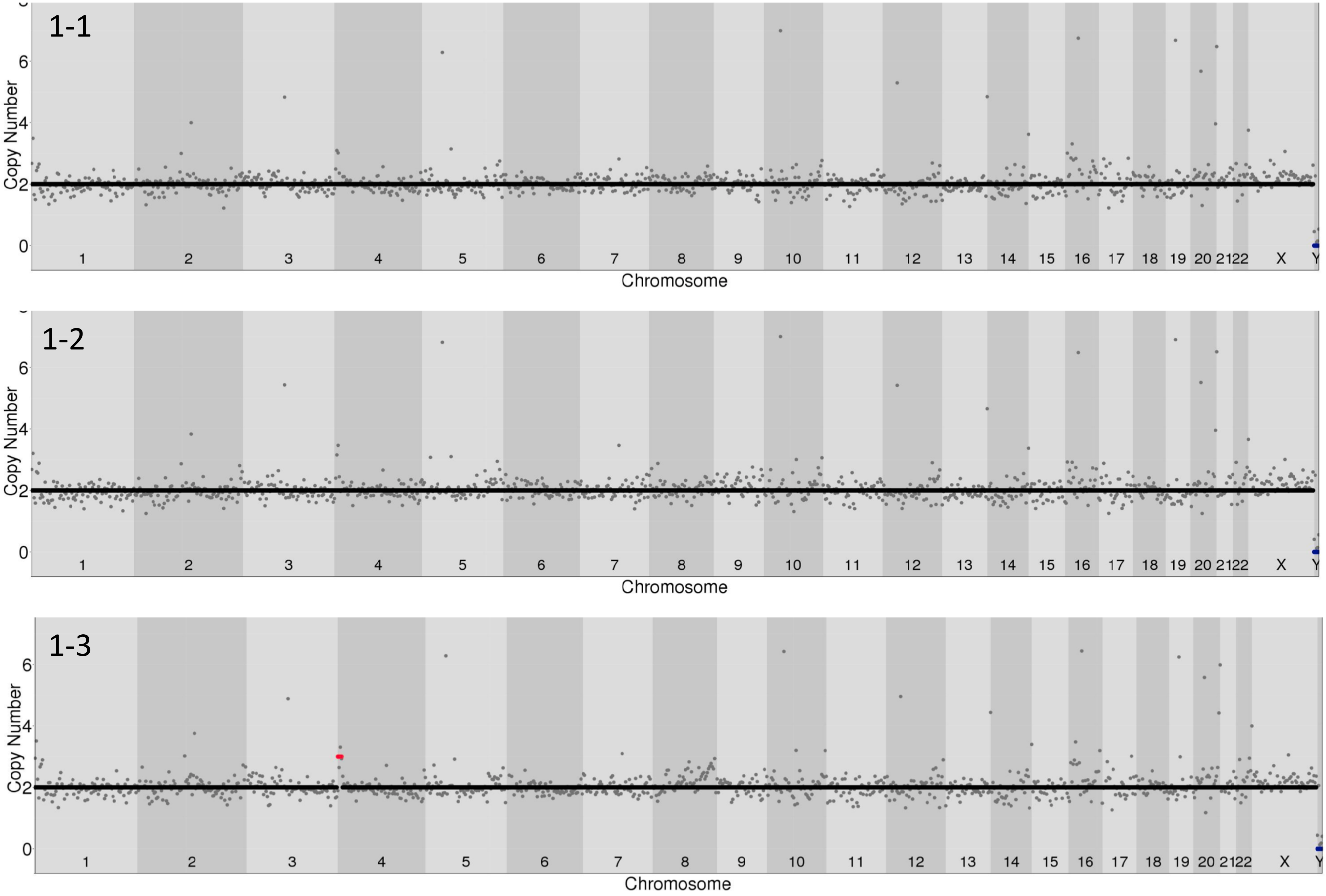

Supplementary Figure S3 (All mock cells)

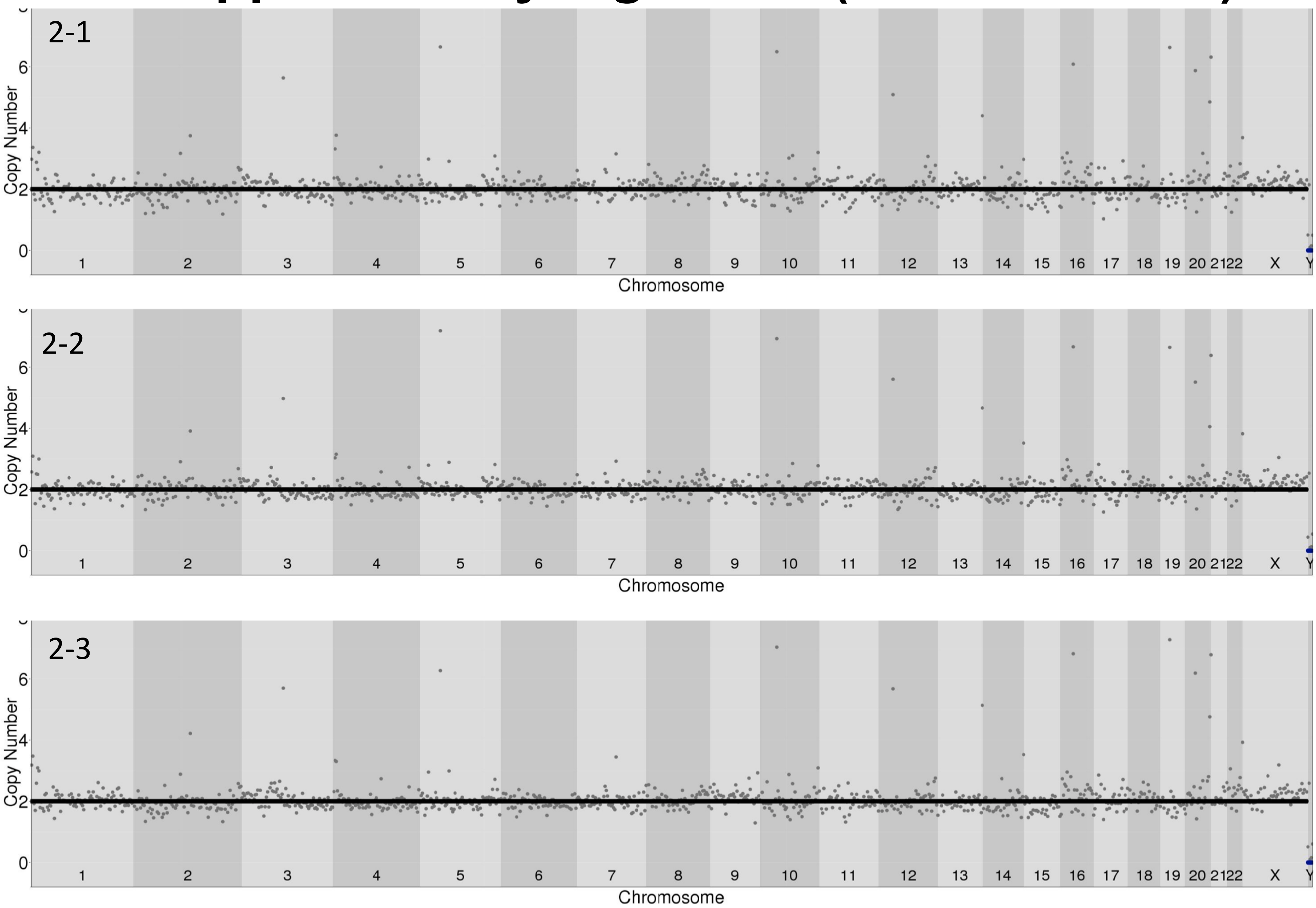

Supplementary Figure S3 (All mock cells)

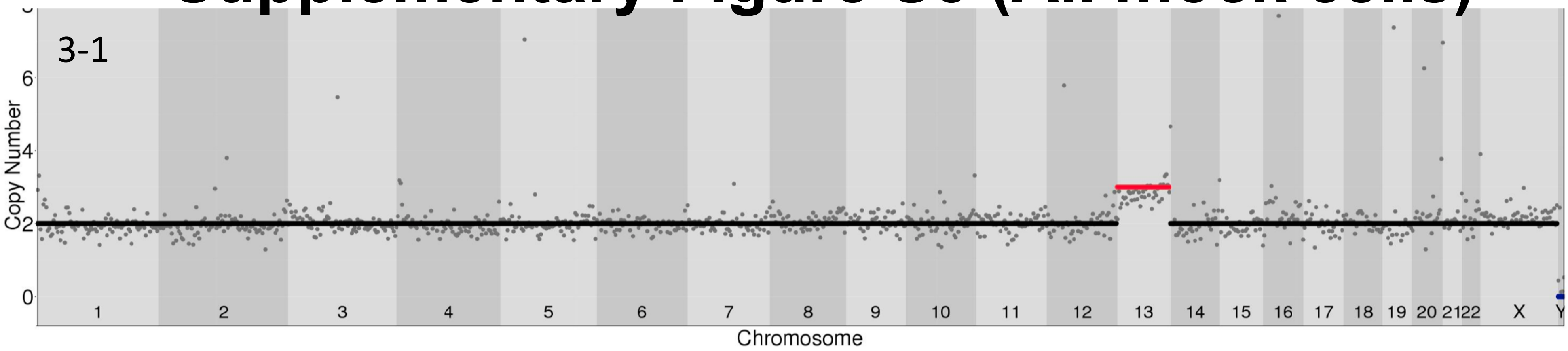

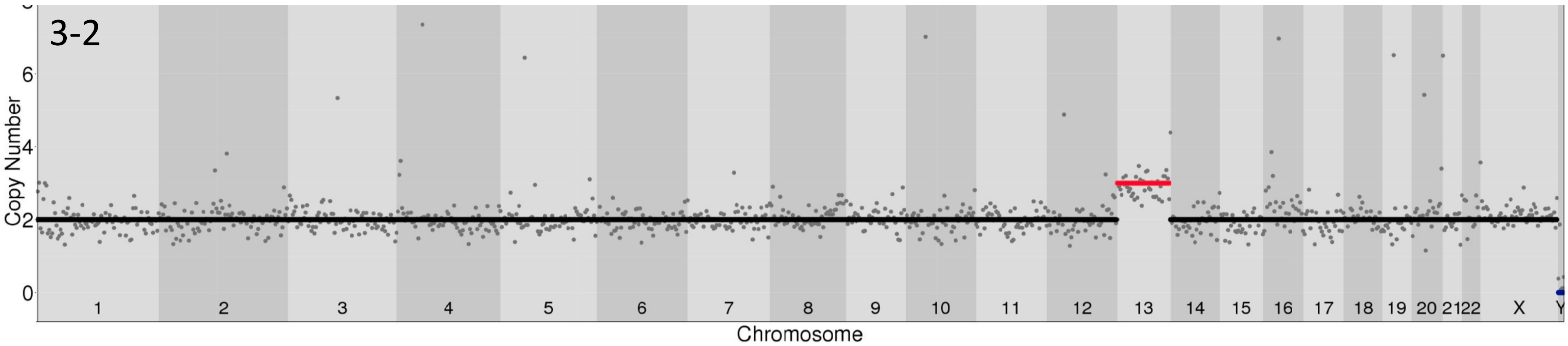

Supplementary Figure S3 (All mock cells)

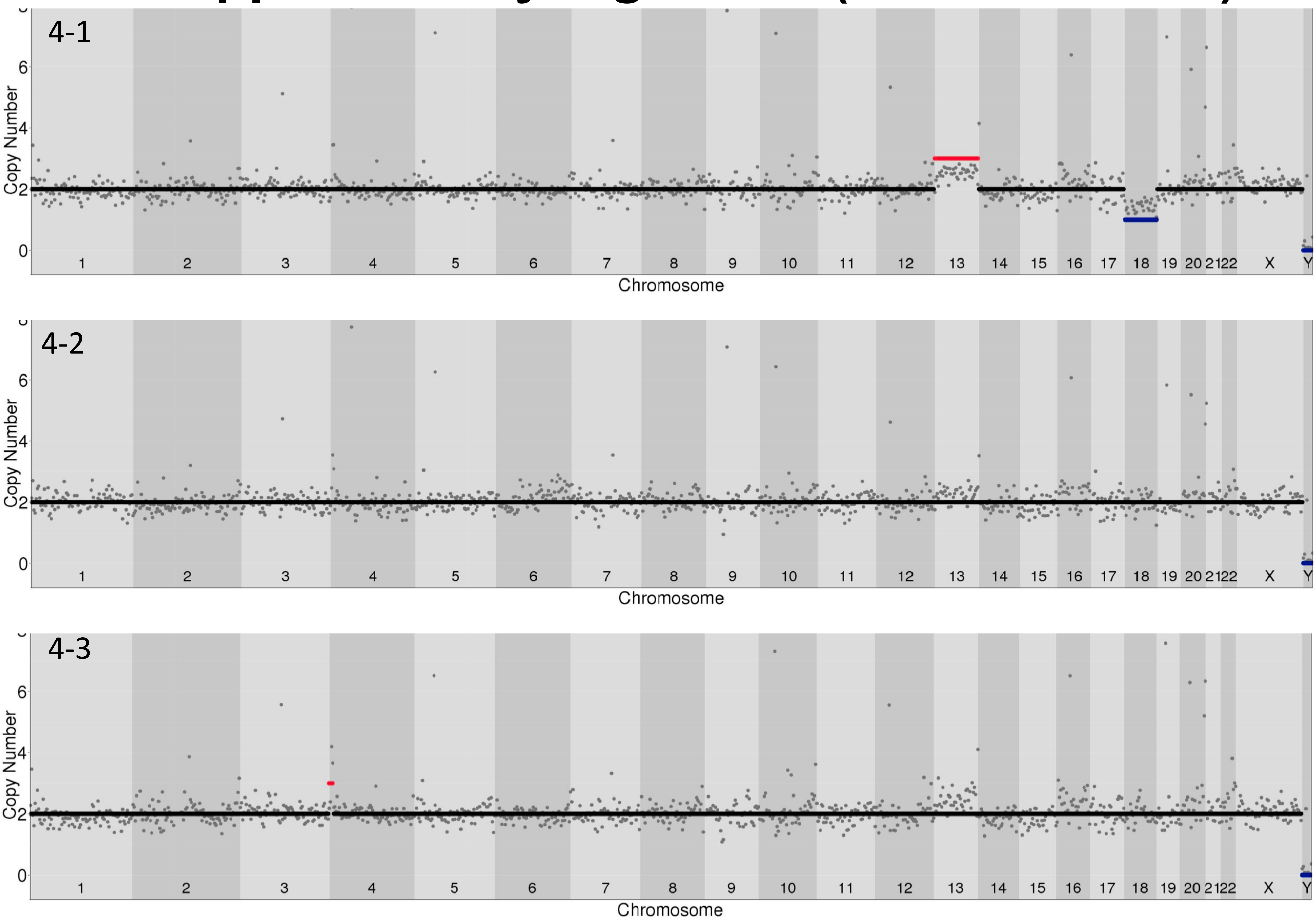

Supplementary Figure S3 (All mock cells)

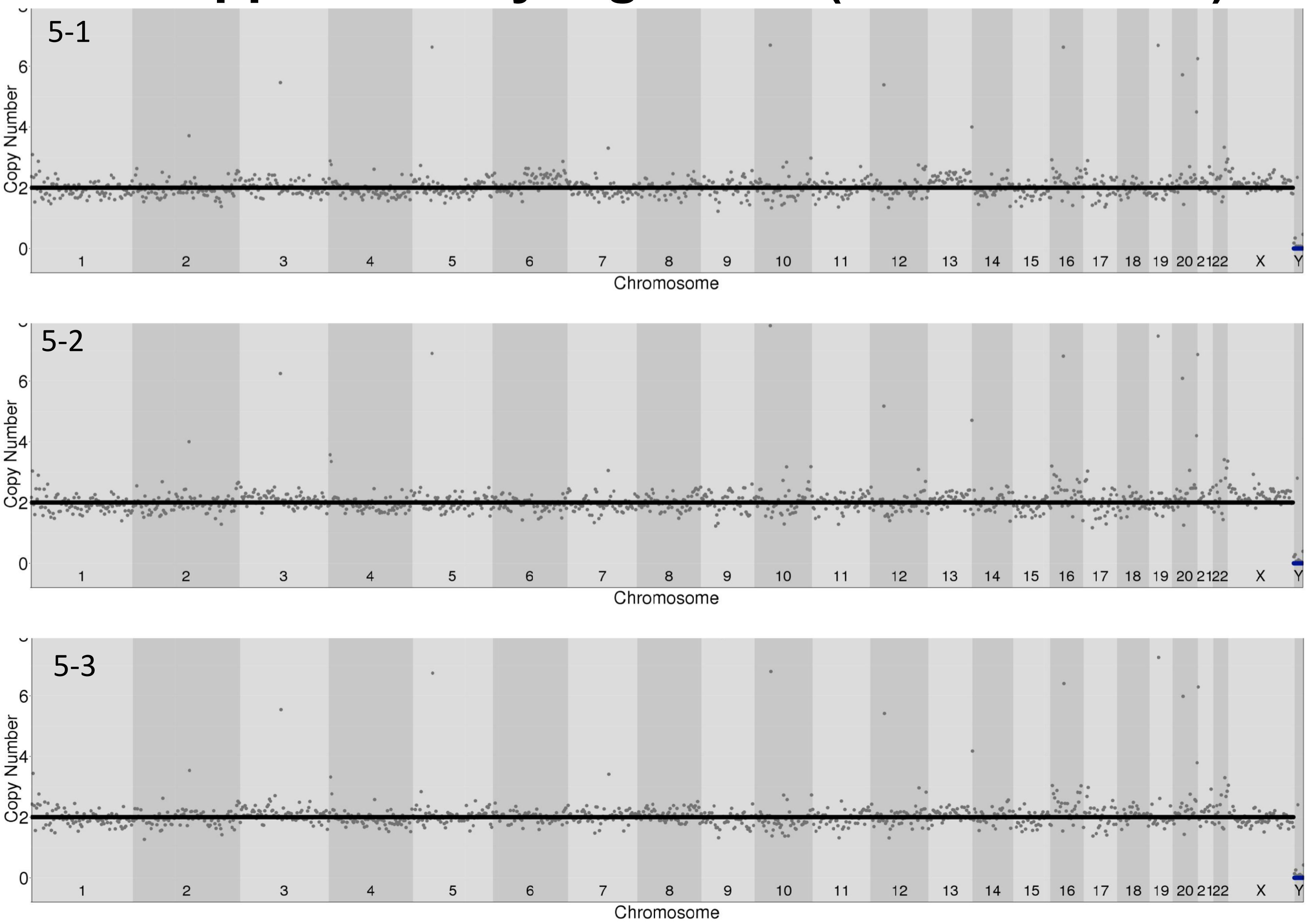

Supplementary Figure S3 (All mock cells)

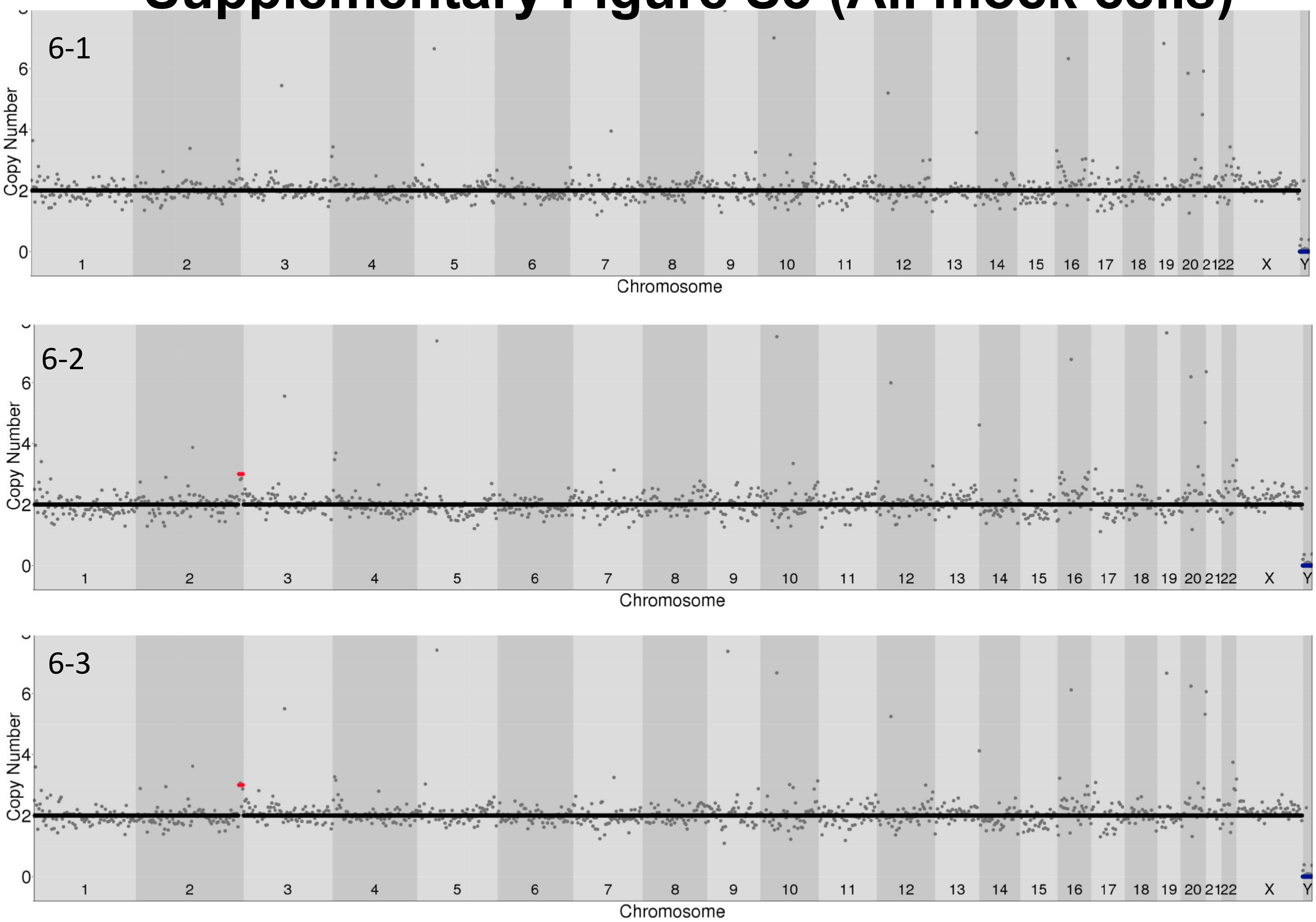

Supplementary Figure S3 (All mock cells)

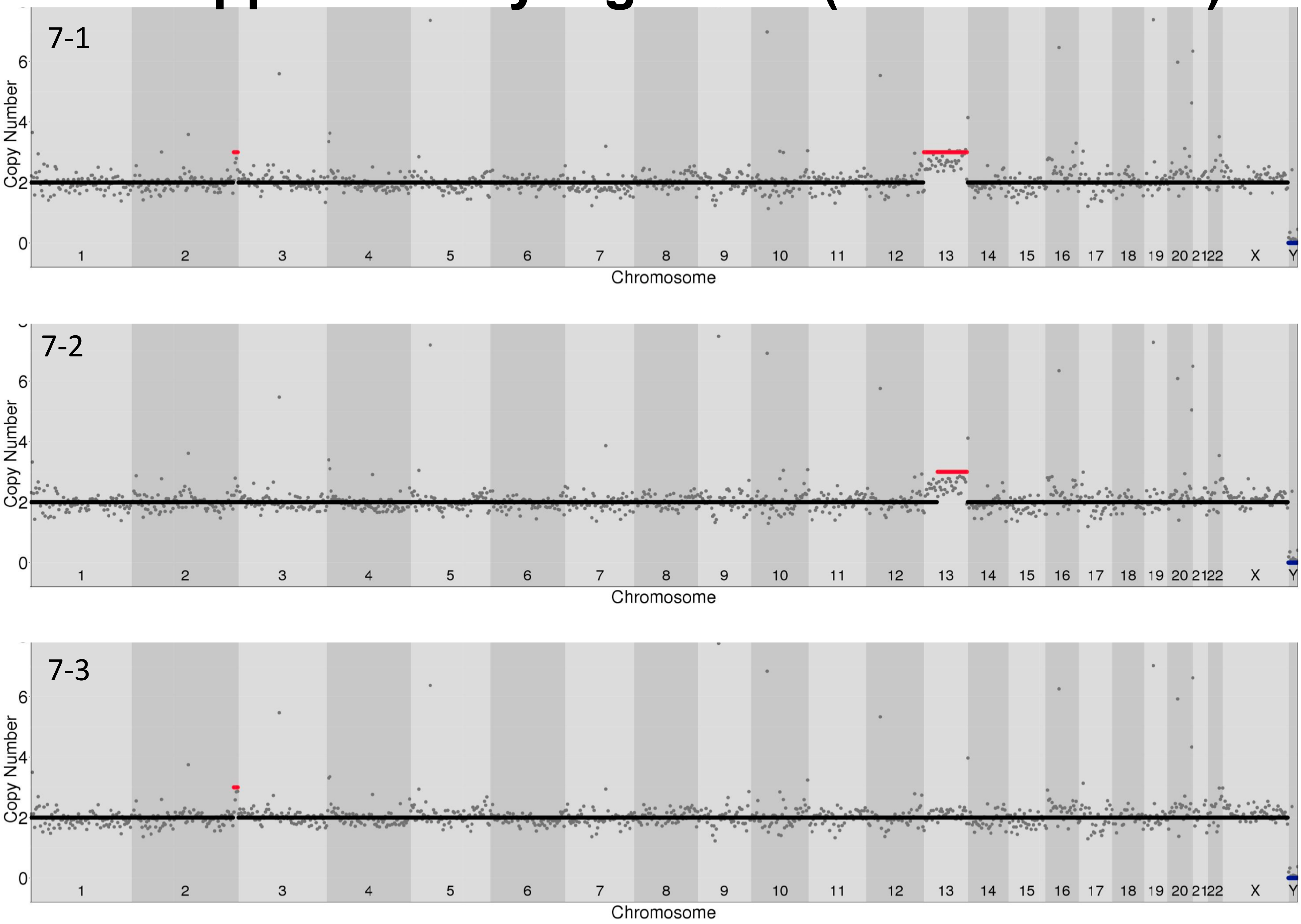

Supplementary Figure S3 (All mock cells)

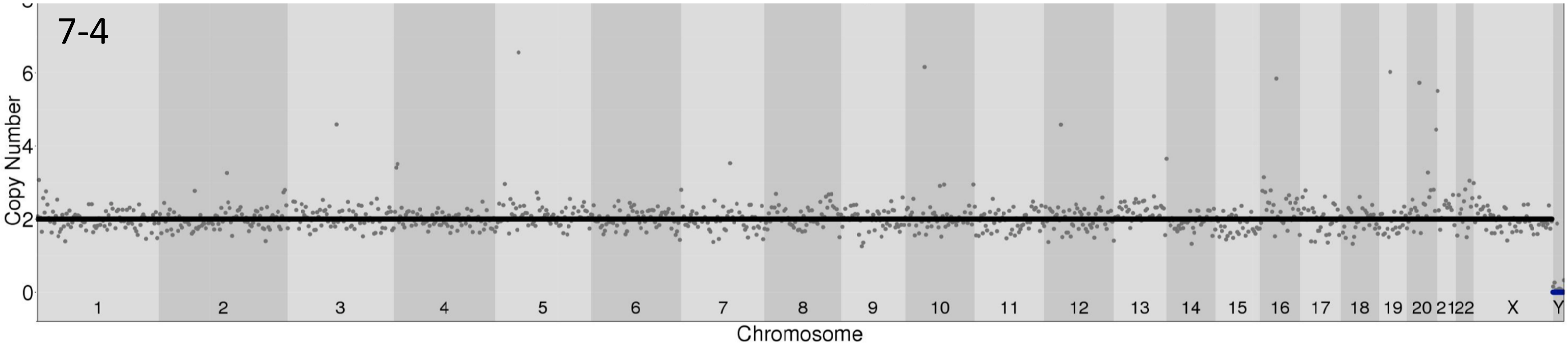

Supplementary Figure S4

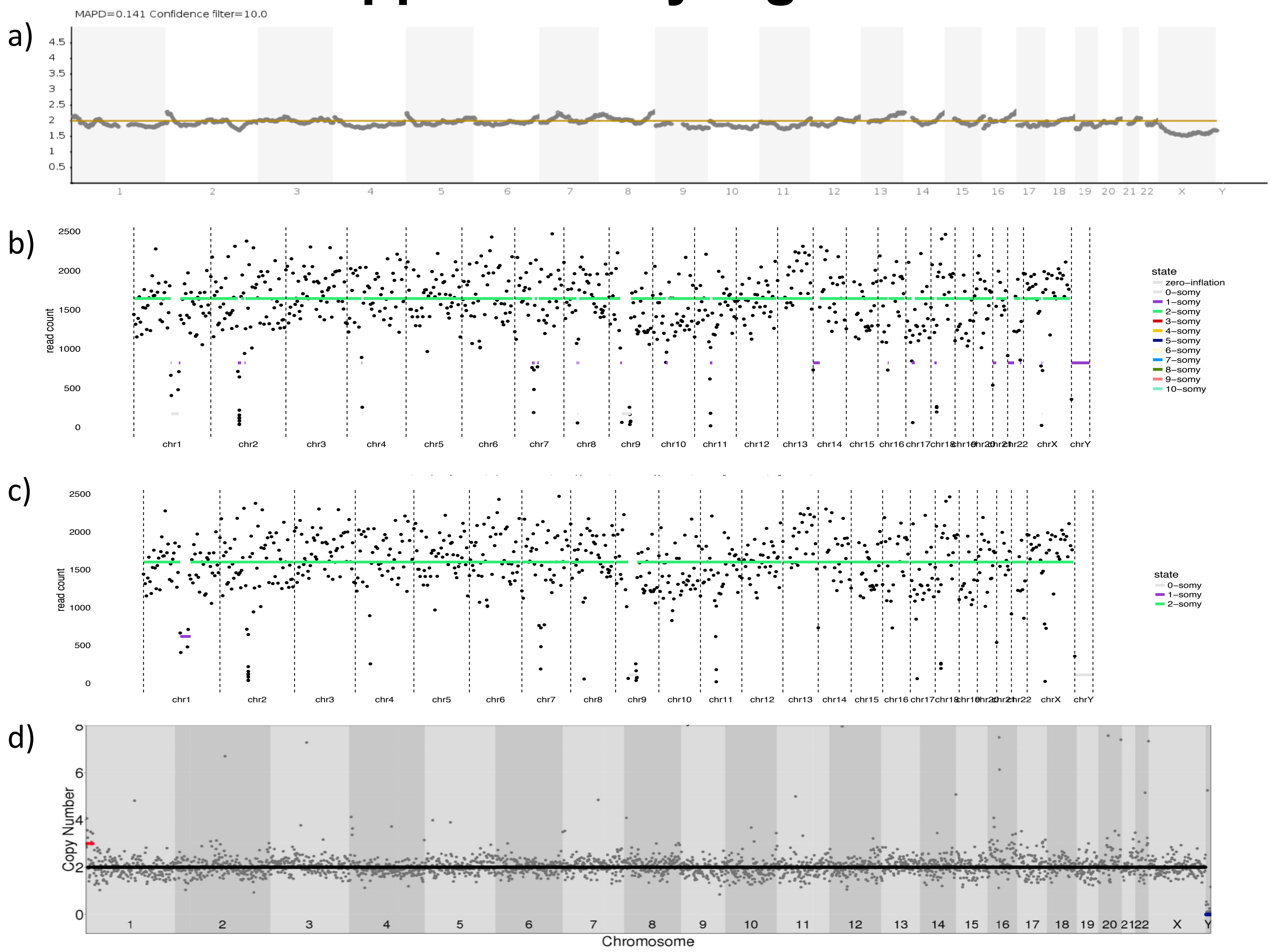

Supplementary Figure S5

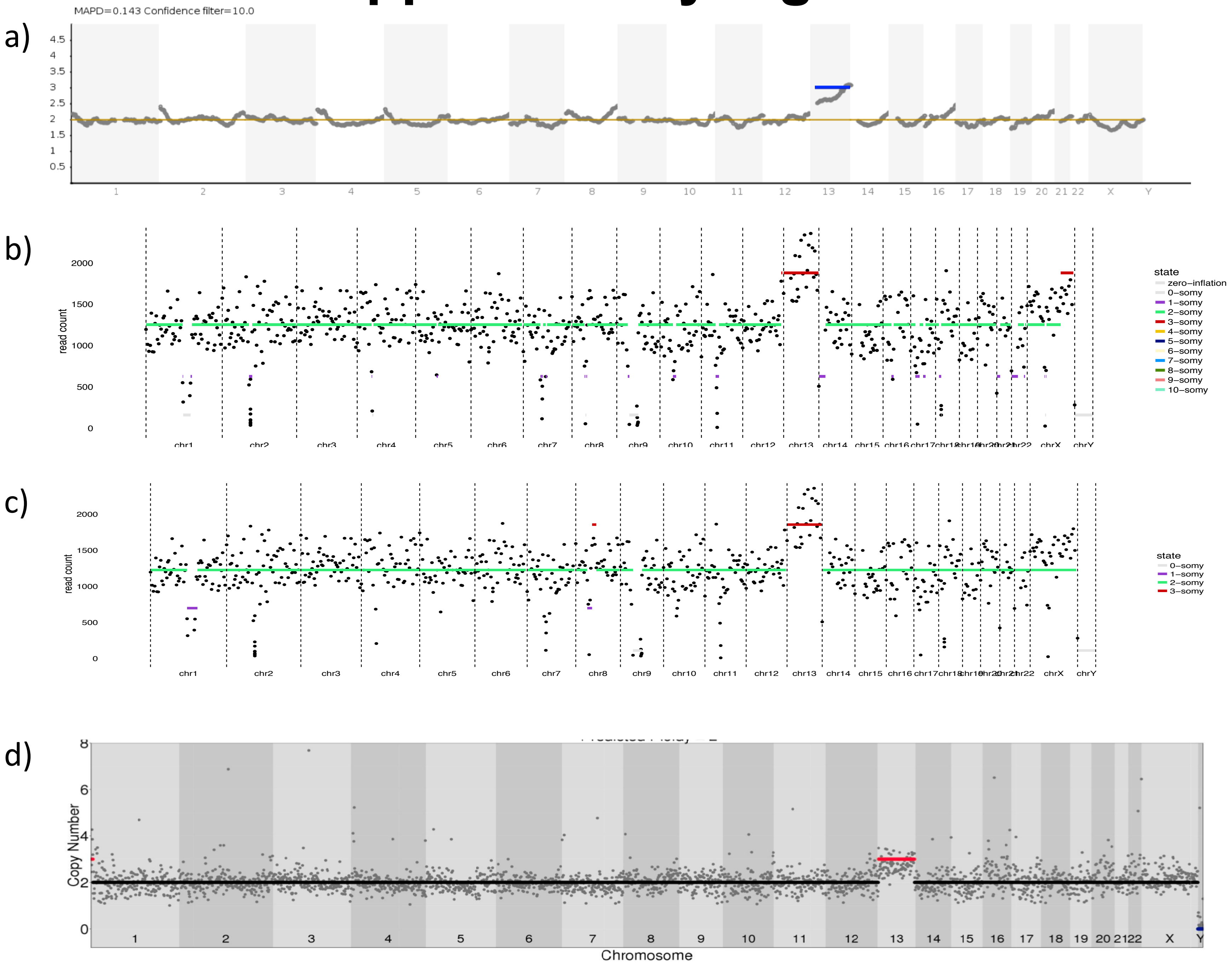

Supplementary Figure S6

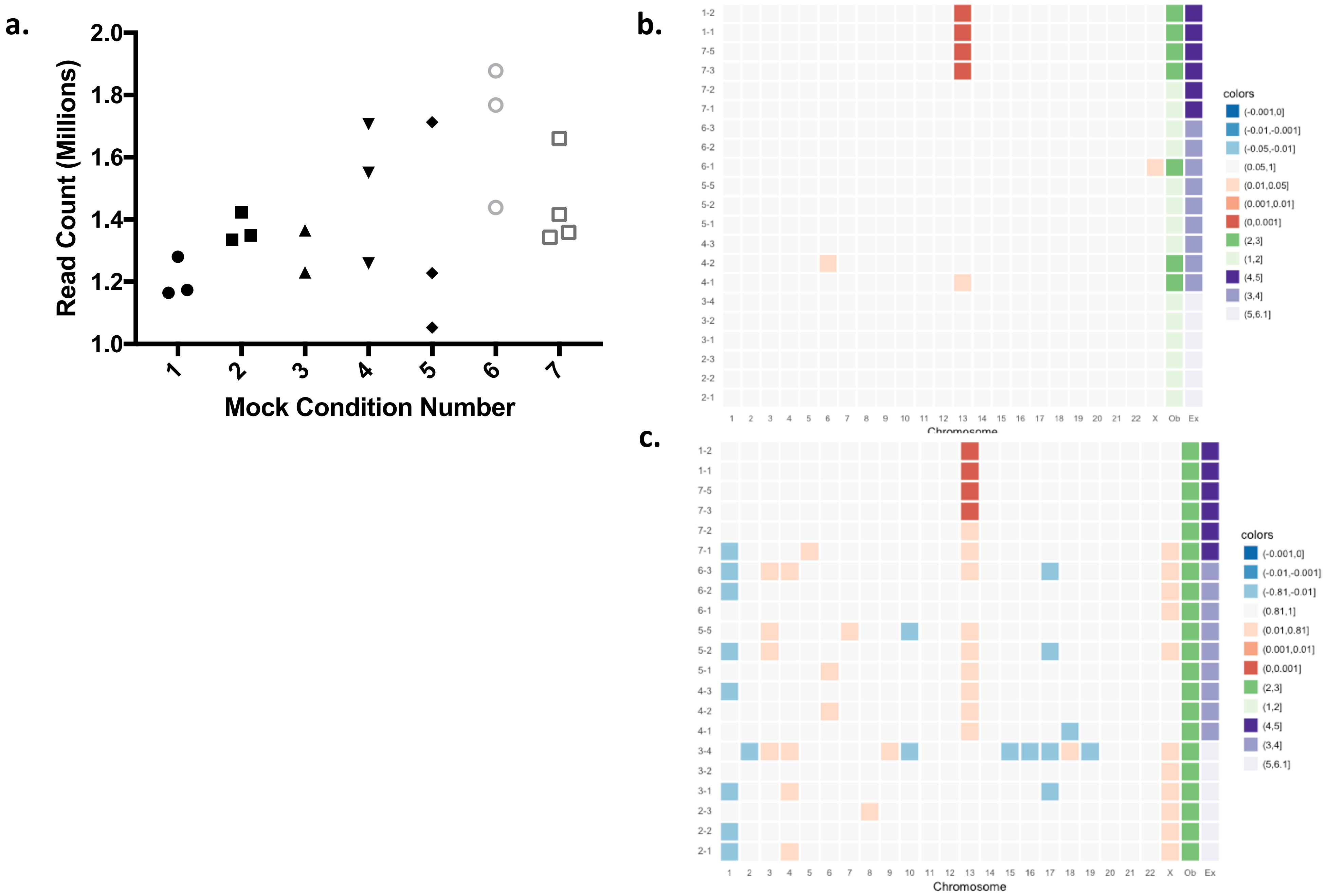

Supplementary Figure S7

PRO1

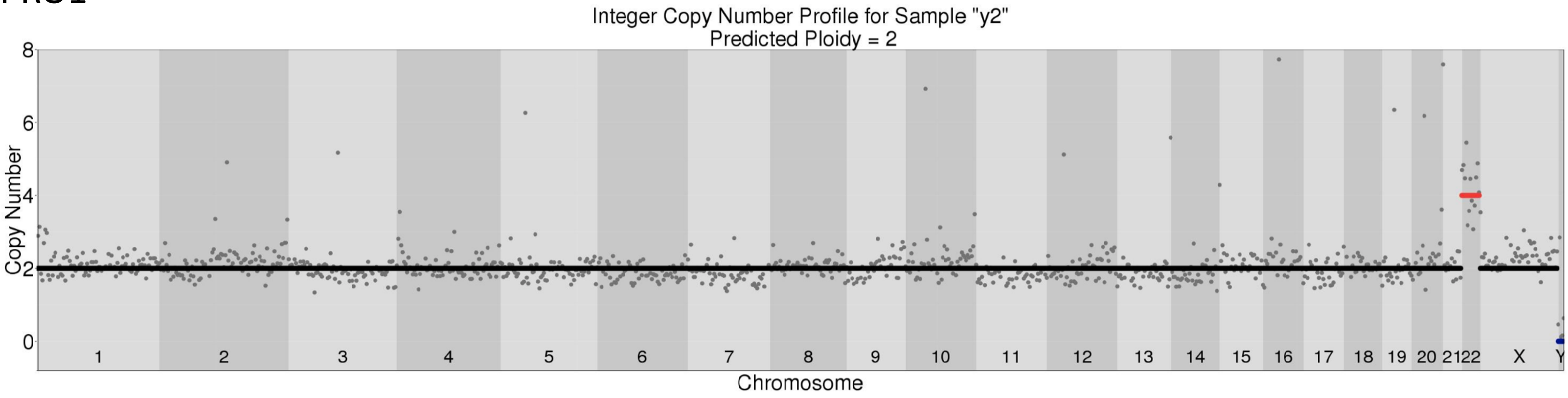

Supplementary Figure S8 (All Not 2n SEN cells)

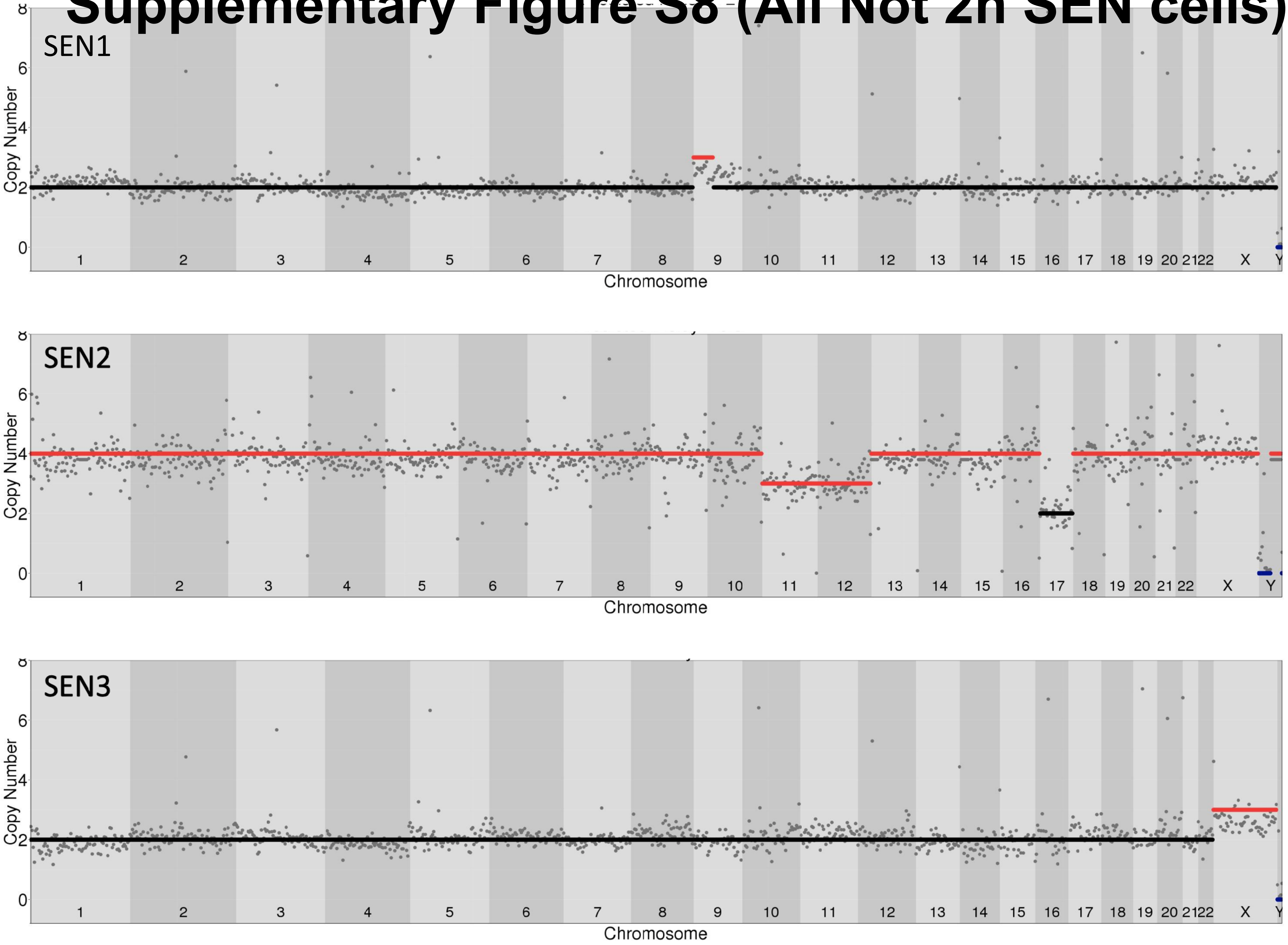

Supplementary Figure S8 (All Not 2n SEN cells)

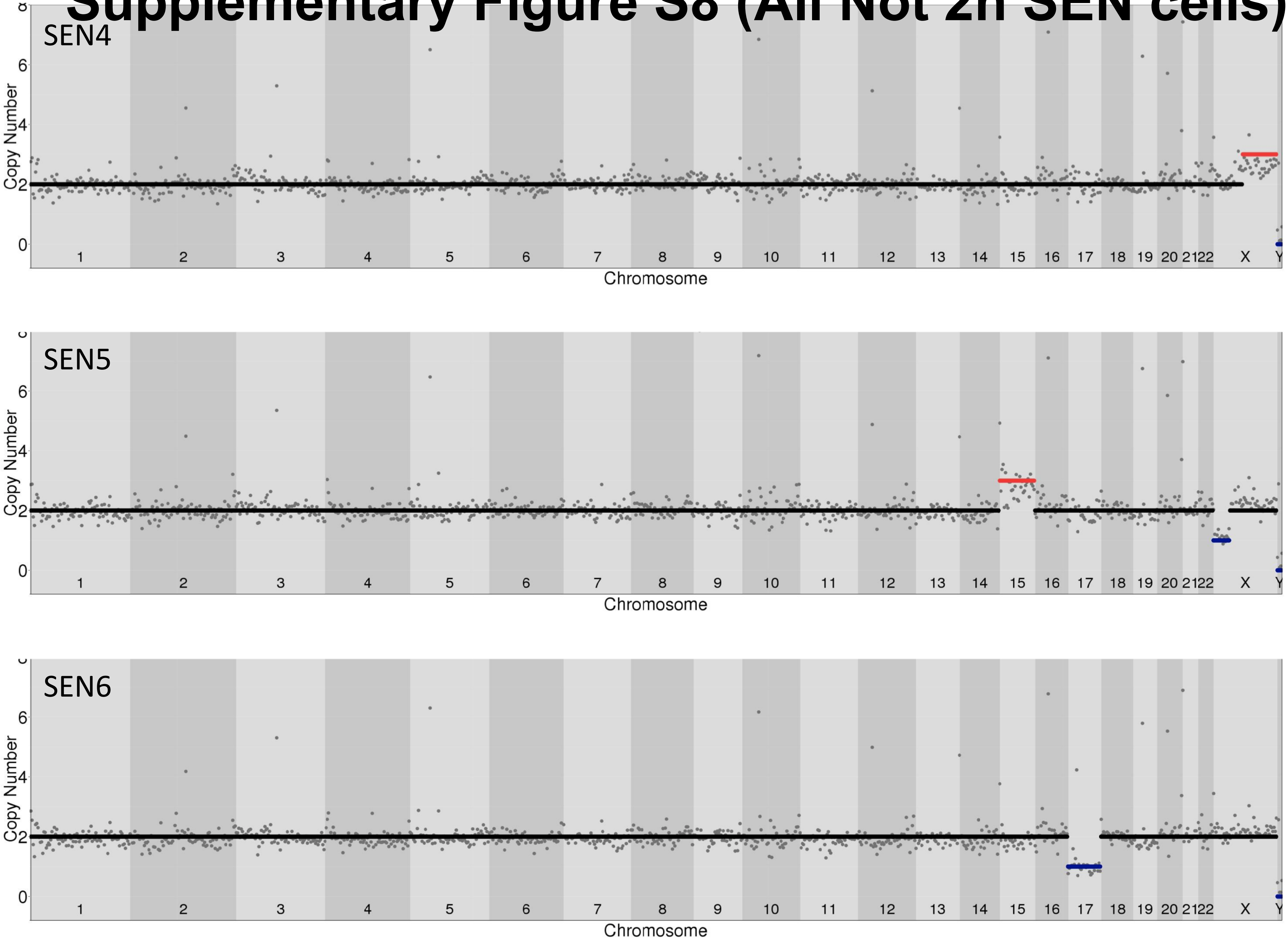

Supplementary Figure S8 (All Not 2n SEN cells)

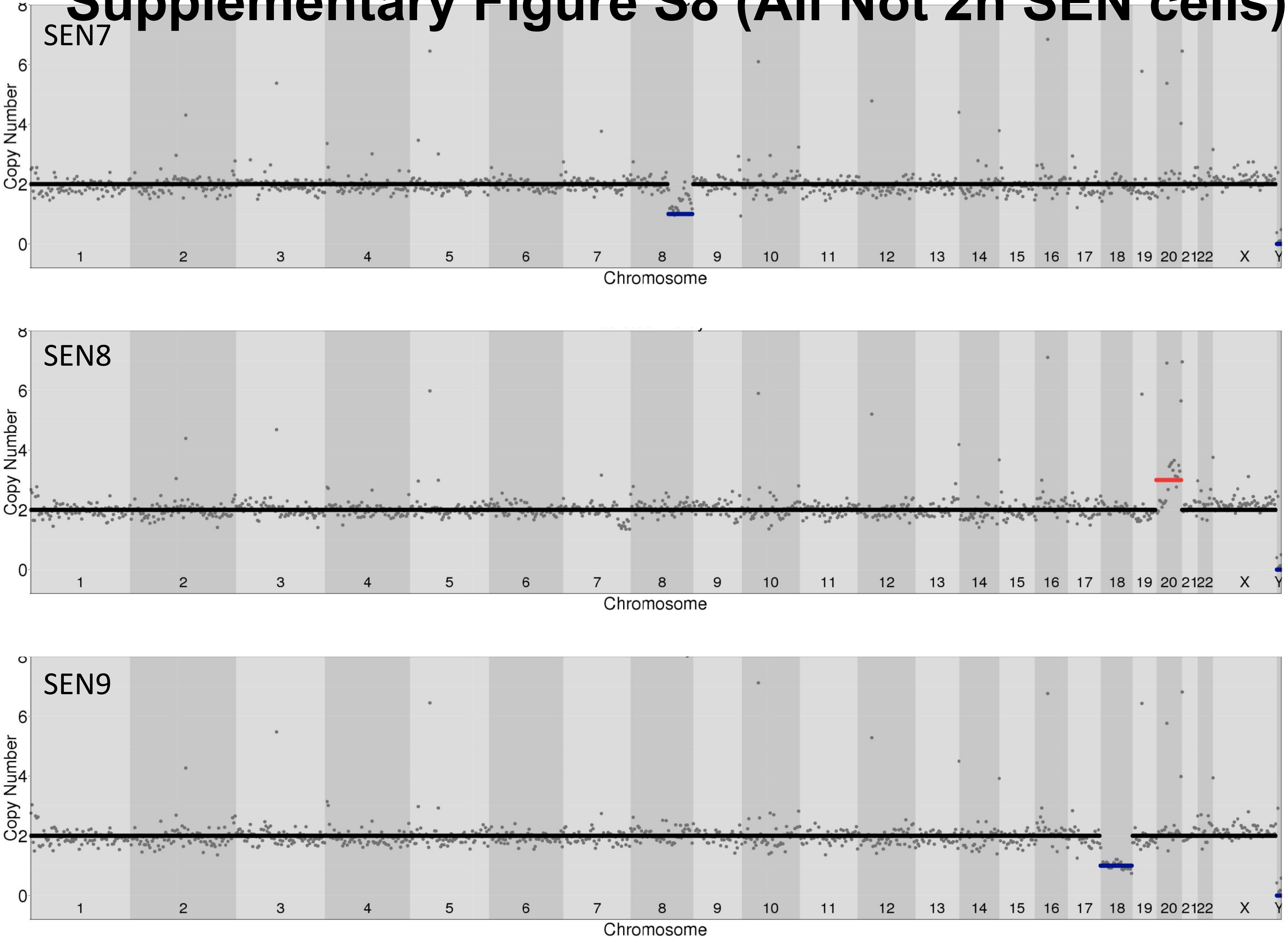

Supplementary Figure S8 (All Not 2n SEN cells)

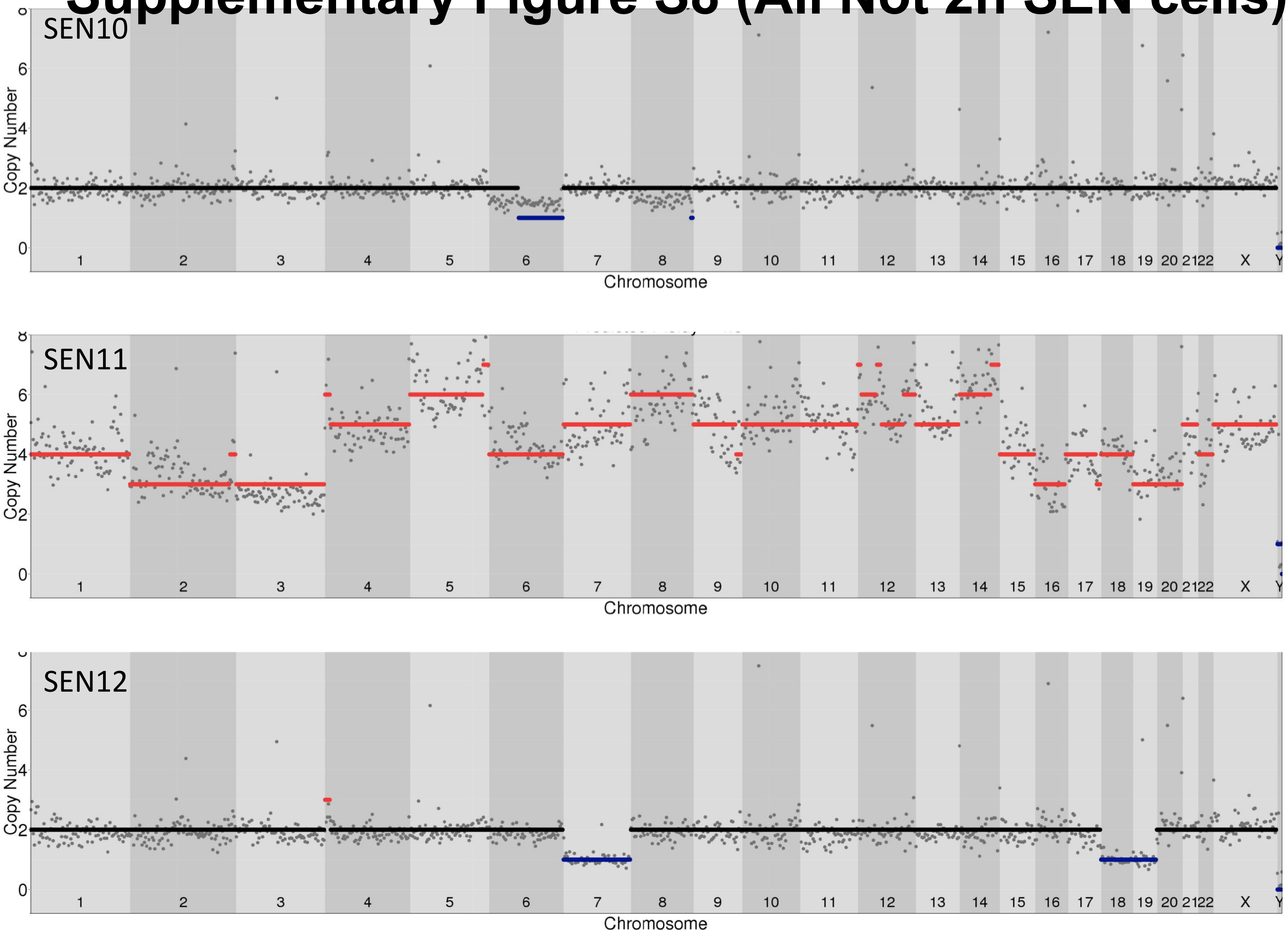

Supplementary Figure S8 (All Not 2n SEN cells)

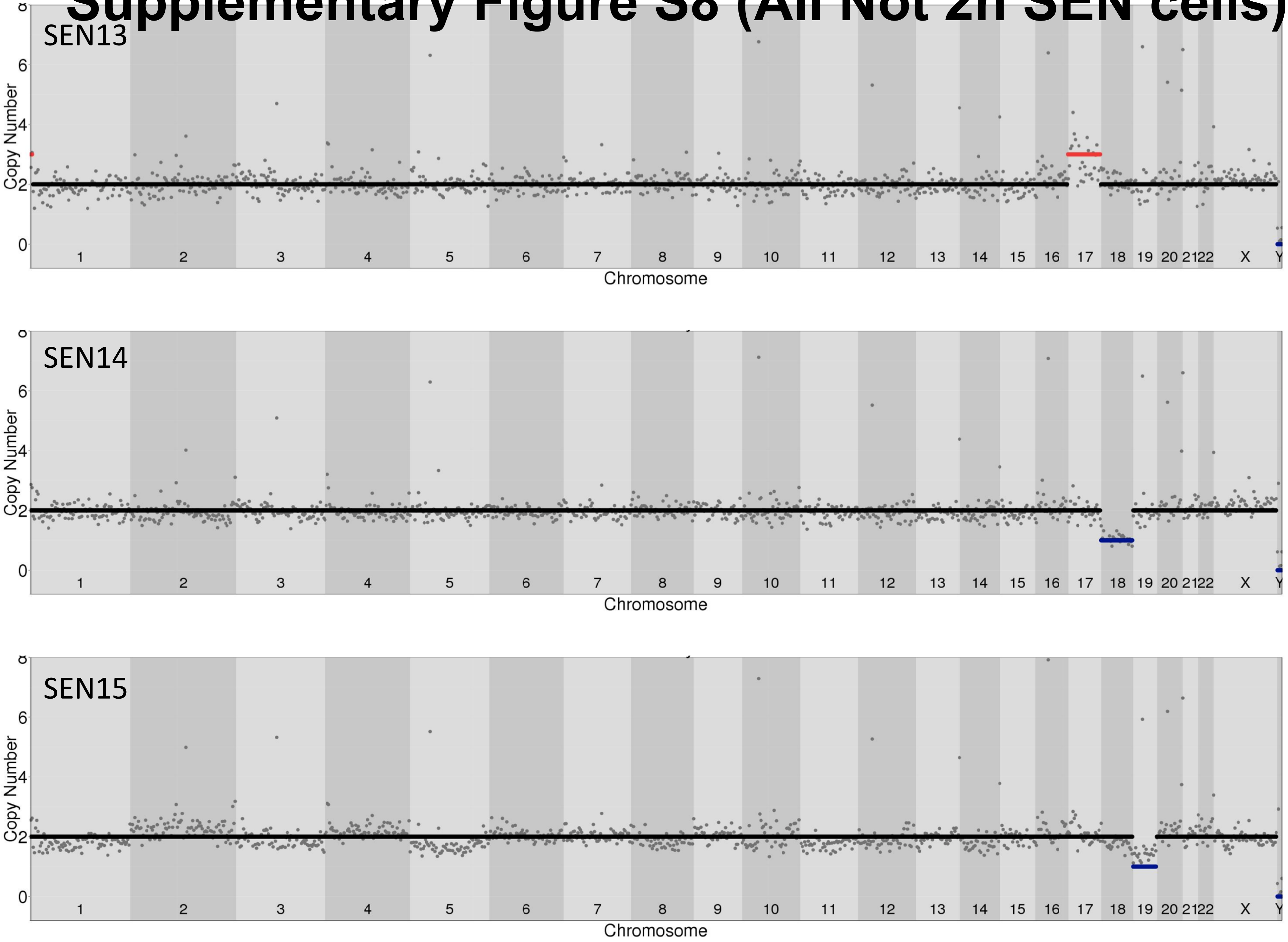

Supplementary Figure S8 (All Not 2n SEN cells)

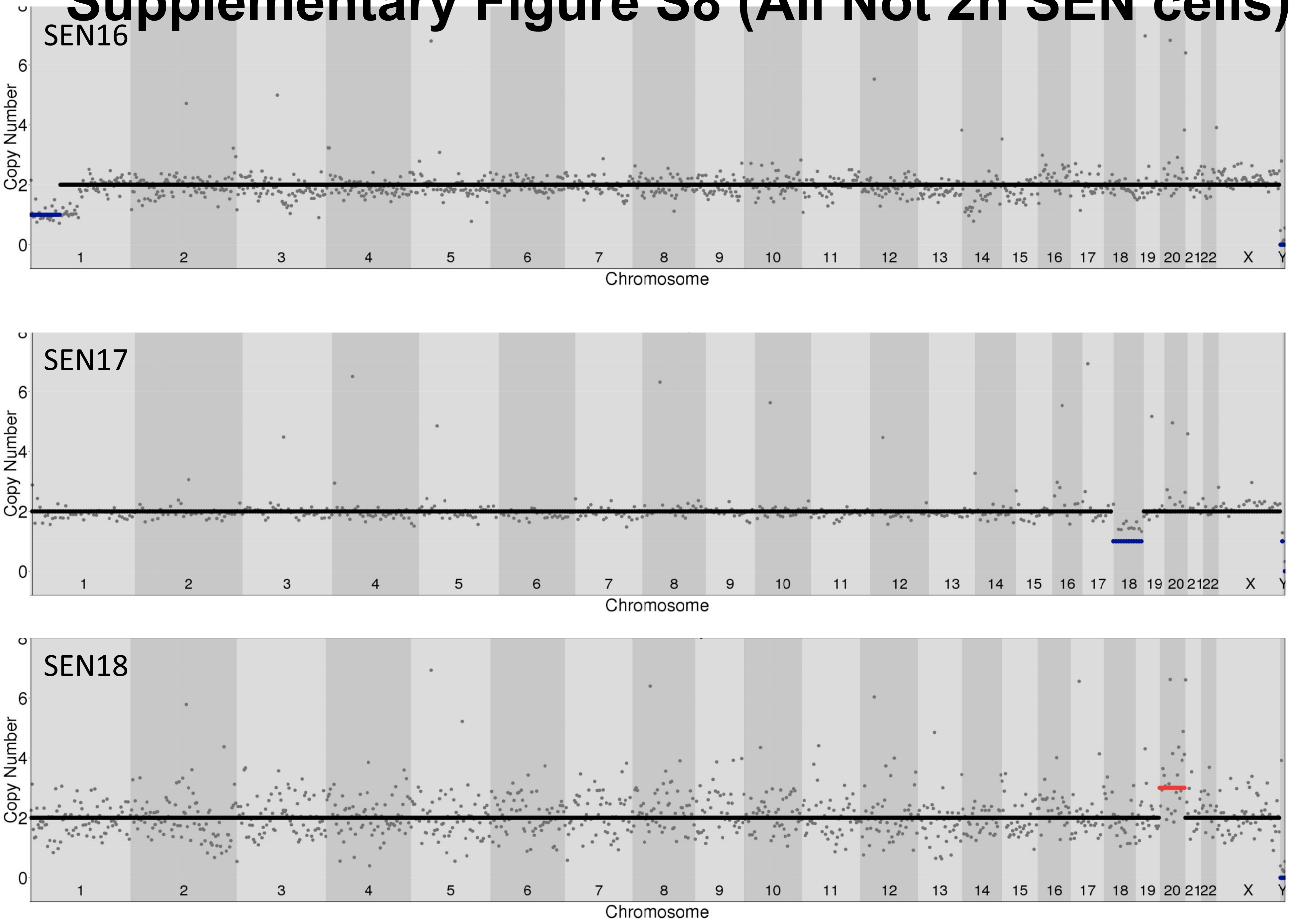

Supplementary Figure S8 (All Not 2n SEN cells)

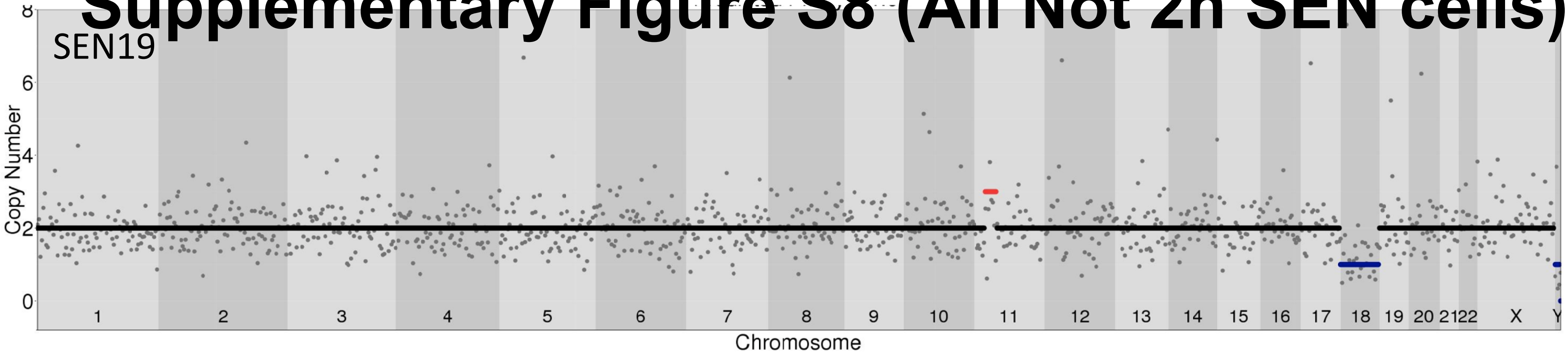

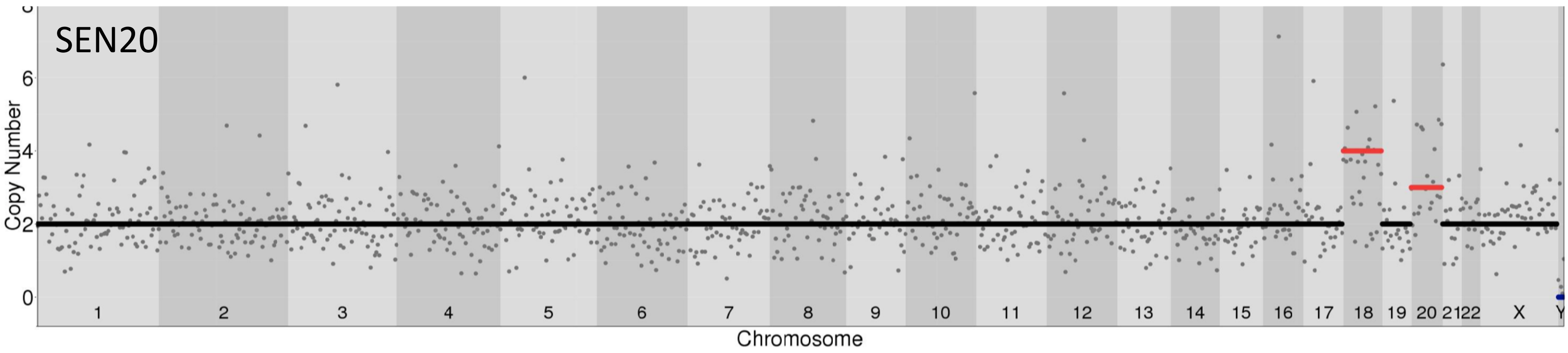

Supplementary Figure S9

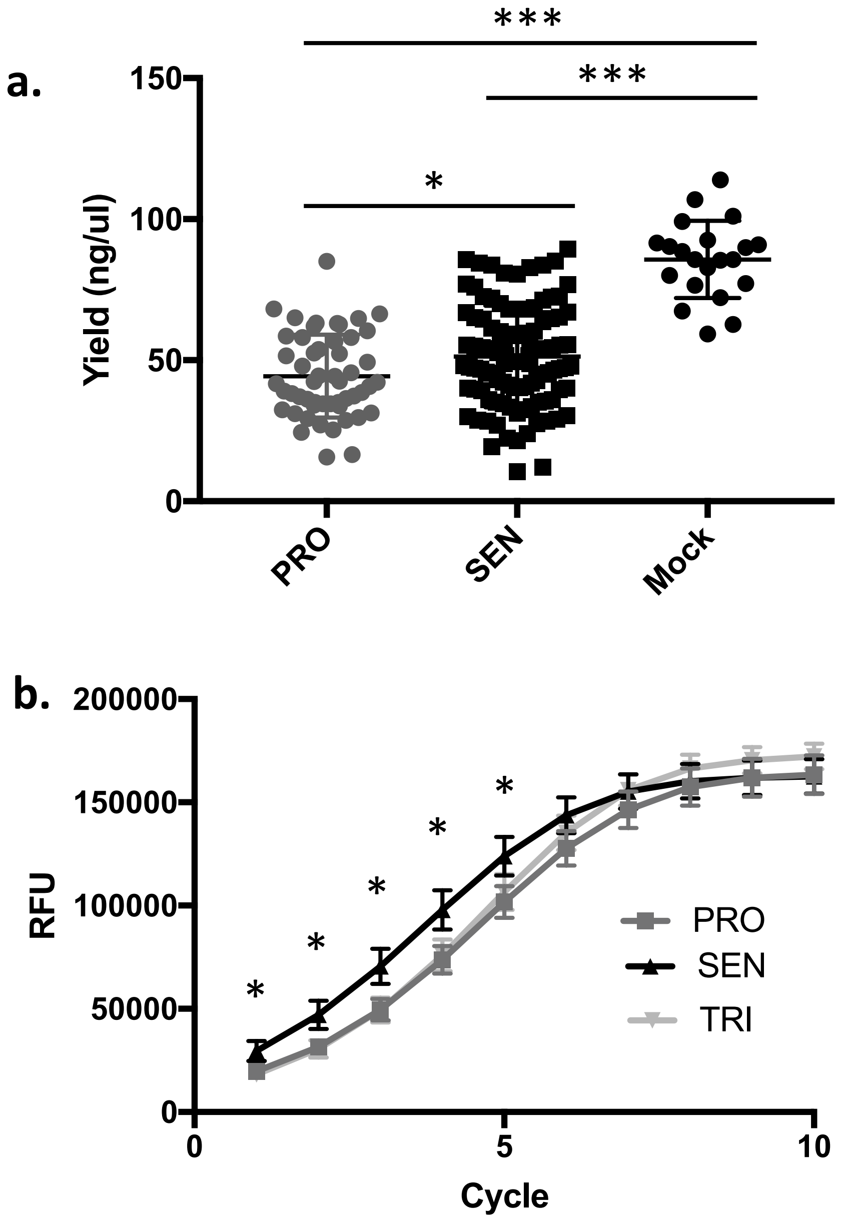

Supplementary figure legends

Supplementary Figure S1 -- Representative Ginkgo copy number graphs are shown for each type of trisomy cell (T13, T18, T21). Each dot represents the copy number of a 2.5 Mb bin, with the line indicating assigned ploidy. Red lines indicate a chromosomal gain and blue lines show a chromosomal loss.

Supplementary Figure S2 -- Validation of the scL-WGS approach. **(a)** Ploidy distribution detected by scL-WGS on T13, T18 or T21 primary fibroblasts. **(b)** Quantification of FISH analysis of T18 metaphase spreads, revealing cells with 1, 2 or 4 copies of chromosome 18. **(c-d)** Representative images of T18 metaphase spreads hybridized with a chromosome painting probe specific for chromosome 18, showing a cell with **(c)** 3 copies and **(d)** 2 copies of chromosome 18.

Supplementary Figures S3 -- Ginkgo copy number graphs are shown for each of the mock aneuploidy conditions generated in this study (depicted in Figure 1). Each dot represents the copy number of a 2.5 Mb bin, with the line indicating assigned ploidy. Red lines indicate a chromosomal gain and blue lines show a chromosomal loss.

Supplementary Figures S4-S5 -- Representative images for two mock aneuploid cells (S4: 1 trisomy 13, 2 IMR90; S5: 2 trisomy 13, 2 IMR90) showing chromosomal copy number calls using **(a)** Ion Reporter suite, **(b)** Aneufinder with HMM, **(c)** Aneufinder using DNAcopy modeling, and **(d)** Ginkgo. All programs yielded the same ploidy calls. Identical binning of 1 Mb was used for Aneufinder and Ginkgo; standard program settings were used for Ion Reporter.

Supplementary Figures S6 -- Failure of computational methods to correctly categorize mock cells consisting of different ploidy (N=21). **(a)**The ploidy of each cell is labeled along the X axis. 5 of the 7 mock ploidy conditions consist of an extra copy of chromosome 13, and the number of chromosome 13 copies is indicated to the right of the underscore within the label (if present). **(b-c)** Plot of the assigned chromosome number for each mock aneuploidy cell populations using our new method of whole chromosome binning with a p value of 0.05 **(b)** or 0.71 **(c)**. Purple boxes indicate expected ploidy for chromosome 13 and dark green boxes indicate ploidy changes in the cell.

Supplementary Figure S7-S8

Ginkgo copy number graphs are shown for each of the PRO (S7) or SEN (S8) cells that was called as aneuploidy. Each dot represents the copy number of a 2.5 Mb bin, with the line indicating assigned ploidy. Red lines indicate a chromosomal gain and blue lines show a chromosomal loss.

Supplementary Figure S9 -- WGA amplification curves and yield differences among different cells types. **(a)** DNA concentration after WGA of PRO (n=48), SEN (n=102), and mock aneuploid cells (n=21). Average yield for SEN was significantly greater than the PRO yield and mock aneuploidy yield was significantly higher than both SEN and PRO cells. * $p<0.05$, ** $p<0.01$, *** $p<0.001$. **(b)** WGA was performed with SYBR dye to measure the relative fluorescent units (RFU) per cycle for PRO (n=14), SEN (n=15), and trisomy cells (n=10). SEN cells had a significantly higher DNA content only at low amplification cycles.

Supplementary Table S1: identity and mapping of Locus Specific Probes (LPS) used in this study

Species	Chromosome	Mapping	bp mapping on chromosome	BAC ID
**Mouse**	1	1qA1	chr1:4,663,133-4,880,417	RP23-34K7
		1qA2	chr1:9,598,485-9,778,150	RP23-102B11
	7	7qA1	chr7:10,264,566-10,497,398	RP23-336P12
		7qF5	chr7:152,331,071-152,515,971	RP24-316N16
	18	18qA2	chr18:23,493,772-23,677,644	RP23-337K12
		18qE2	chr18:66,990,219-67,213,556	RP23-16K15
**Human**	9	9q21.12	chr9:73,923,178-74,091,305	RP11-8L13
		9p13.3	chr9:34,687,551-34,830,126	RP11-18K11
	12	12p12.1	chr12:25,872,059-26,035,756	RP11-51C9
		12q14.1	chr12:60,446,361-60,631,044	RP11-35G5

Supplementary Table S2: comparison between FISH signals obtained by 1 or 2 probe/chromosome

FISH approach	1 probe/chromosome FISH	2 probe/chromosome FISH
% Not 2n cells	1.7	0.9
	1.7	0.7
	3.0	2.0
	2.7	0.8
	-	0.6
	-	0.6
**Average**	**2.3**	**0.9**
**SD**	0.7	0.6

Supplementary Table S3: Ginkgo settings tested for mock aneuploid conditions

Settings	Gingko settings	Correct ploidy (%)	Correct aneuploidy (%)	False positive (%)	False positive or negative calls in 2n+13/2n+18/2n+21
**1**	500Kb fixed window Global normalization	0	28.6	4.8	YES
**2**	500Kb fixed window Independent normalization	0	28.6	4.8	YES
**3**	1Mb variable window Global normalization	0	28.6	4.8	YES
**4**	1Mb variable window Independent normalization	0	28.6	4.8	YES
**5**	1Mb fixed window Global normalization	0	28.6	9.5	-
**6**	1Mb fixed window Independent normalization	0	28.6	9.5	-
**7**	500Kb variable window Global normalization	0	23.8	4.8	YES
**8**	500Kb variable window Independent normalization	0	23.8	4.8	YES
**9**	**2.5Mb variable window Global normalization**	**0**	**23.8**	**4.8**	**NO**
**10**	2.5Mb fixed window Global normalization	0	23.8	4.8	YES
**11**	2.5Mb fixed window Independent normalization	0	23.8	4.8	YES
**12**	2.5Mb variable window Independent normalization	0	19	9.5	-
**13**	10Mb fixed window Global normalization	0	19	4.8	-
**14**	10Mb fixed window Independent normalization	0	19	4.8	-

Supplementary Table S4: iFISH counts per cell

PRO			SEN		
Cell number	Copy number of Ch. 12	Copy number of Ch. 9	Cell number	Copy number of Ch. 12	Copy number of Ch. 9
Cell 1	8	8	Cell 1	17	15
Cell 2	8	8	Cell 2	16	16
Cell 3	8	8	Cell 3	16	16
Cell 4	8	8	Cell 4	16	12
Cell 5	8	8	Cell 5	15	16
Cell 6	5	2	Cell 6	14	16
Cell 7	5	2	Cell 7	14	14
Cell 8	4	4	Cell 8	13	16
Cell 9	4	4	Cell 9	12	12
Cell 10	4	4	Cell 10	12	12
Cell 11	4	4	Cell 11	10	8
Cell 12	4	4	Cell 12	10	8
Cell 13	4	4	Cell 13	9	15
Cell 14	4	4	Cell 14	9	8
Cell 15	4	4	Cell 15	9	8
Cell 16	4	4	Cell 16	8	10
Cell 17	4	4	Cell 17	8	9
Cell 18	4	4	Cell 18	8	9
Cell 19	4	4	Cell 19	8	8
Cell 20	4	4	Cell 20	8	8
Cell 21	4	4	Cell 21	8	8
Cell 22	4	4	Cell 22	8	8
Cell 23	4	4	Cell 23	8	8
Cell 24	4	4	Cell 24	8	8
Cell 25	4	4	Cell 25	8	8
Cell 26	4	4	Cell 26	8	8
Cell 27	4	4	Cell 27	8	8
Cell 28	4	4	Cell 28	8	8
Cell 29	4	4	Cell 29	8	8
Cell 30	4	4	Cell 30	8	8
Cell 31	4	4	Cell 31	8	8
Cell 32	4	4	Cell 32	8	8
Cell 33	4	4	Cell 33	8	8
Cell 34	4	2	Cell 34	8	8
Cell 35	4	2	Cell 35	8	8
Cell 36	4	2	Cell 36	8	8

Cell 37	4	2	Cell 37	8	8
Cell 38	4	2	Cell 38	8	8
Cell 39	4	2	Cell 39	8	8
Cell 40	3	3	Cell 40	8	8
Cell 41	3	2	Cell 41	8	8
Cell 42	3	2	Cell 42	8	8
Cell 43	3	2	Cell 43	8	8
Cell 44	3	2	Cell 44	8	8
Cell 45	3	2	Cell 45	8	8
Cell 46	3	2	Cell 46	8	8
Cell 47	3	2	Cell 47	8	8
Cell 48	3	2	Cell 48	8	8
Cell 49	2	3	Cell 49	8	8
Cell 50	2	3	Cell 50	8	8
Cell 51	2	3	Cell 51	8	8
Cell 52	2	3	Cell 52	8	8
Cell 53	2	3	Cell 53	8	8
Cell 54	2	3	Cell 54	8	8
Cell 55	2	3	Cell 55	8	8
Cell 56	2	3	Cell 56	8	8
Cell 57	2	2	Cell 57	8	8
Cell 58	2	2	Cell 58	8	8
Cell 59	2	2	Cell 59	8	8
Cell 60	2	2	Cell 60	8	8
Cell 61	2	2	Cell 61	8	8
Cell 62	2	2	Cell 62	8	8
Cell 63	2	2	Cell 63	8	8
Cell 64	2	2	Cell 64	8	8
Cell 65	2	2	Cell 65	8	8
Cell 66	2	2	Cell 66	8	8
Cell 67	2	2	Cell 67	8	7
Cell 68	2	2	Cell 68	8	7
Cell 69	2	2	Cell 69	8	6
Cell 70	2	2	Cell 70	8	6
Cell 71	2	2	Cell 71	8	6
Cell 72	2	2	Cell 72	8	6
Cell 73	2	2	Cell 73	8	4
Cell 74	2	2	Cell 74	8	4
Cell 75	2	2	Cell 75	8	4
Cell 76	2	2	Cell 76	7	8
Cell 77	2	2	Cell 77	7	8
Cell 78	2	2	Cell 78	7	7
Cell 79	2	2	Cell 79	6	8

Cell 80	2	2	Cell 80	6	8
Cell 81	2	2	Cell 81	6	8
Cell 82	2	2	Cell 82	6	8
Cell 83	2	2	Cell 83	6	6
Cell 84	2	2	Cell 84	6	6
Cell 85	2	2	Cell 85	6	6
Cell 86	2	2	Cell 86	6	6
Cell 87	2	2	Cell 87	6	6
Cell 88	2	2	Cell 88	6	6
Cell 89	2	2	Cell 89	6	6
Cell 90	2	2	Cell 90	6	4
Cell 91	2	2	Cell 91	6	4
Cell 92	2	2	Cell 92	6	4
Cell 93	2	2	Cell 93	6	4
Cell 94	2	2	Cell 94	6	4
Cell 95	2	2	Cell 95	6	4
Cell 96	2	2	Cell 96	6	4
Cell 97	2	2	Cell 97	5	6
Cell 98	2	2	Cell 98	5	6
Cell 99	2	2	Cell 99	5	5
Cell 100	2	2	Cell 100	5	5
Cell 101	2	2	Cell 101	5	5
Cell 102	2	2	Cell 102	5	5
Cell 103	2	2	Cell 103	5	4
Cell 104	2	2	Cell 104	5	4
Cell 105	2	2	Cell 105	5	4
Cell 106	2	2	Cell 106	5	4
Cell 107	2	2	Cell 107	5	4
Cell 108	2	2	Cell 108	5	4
Cell 109	2	2	Cell 109	5	4
Cell 110	2	2	Cell 110	5	2
Cell 111	2	2	Cell 111	5	2
Cell 112	2	2	Cell 112	4	4
Cell 113	2	2	Cell 113	4	9
Cell 114	2	2	Cell 114	4	8
Cell 115	2	2	Cell 115	4	8
Cell 116	2	2	Cell 116	4	8
Cell 117	2	2	Cell 117	4	8
Cell 118	2	2	Cell 118	4	8
Cell 119	2	2	Cell 119	4	7
Cell 120	2	2	Cell 120	4	7
Cell 121	2	2	Cell 121	4	6
Cell 122	2	2	Cell 122	4	6

Cell 123	2	2	Cell 123	4	6
Cell 124	2	2	Cell 124	4	6
Cell 125	2	2	Cell 125	4	6
Cell 126	2	2	Cell 126	4	5
Cell 127	2	2	Cell 127	4	5
Cell 128	2	2	Cell 128	4	5
Cell 129	2	2	Cell 129	4	5
Cell 130	2	2	Cell 130	4	5
Cell 131	2	2	Cell 131	4	5
Cell 132	2	2	Cell 132	4	5
Cell 133	2	2	Cell 133	4	5
Cell 134	2	2	Cell 134	4	5
Cell 135	2	2	Cell 135	4	5
Cell 136	2	2	Cell 136	4	5
Cell 137	2	2	Cell 137	4	4
Cell 138	2	2	Cell 138	4	4
Cell 139	2	2	Cell 139	4	4
Cell 140	2	2	Cell 140	4	4
Cell 141	2	2	Cell 141	4	4
Cell 142	2	2	Cell 142	4	4
Cell 143	2	2	Cell 143	4	4
Cell 144	2	2	Cell 144	4	4
Cell 145	2	2	Cell 145	4	4
Cell 146	2	2	Cell 146	4	4
Cell 147	2	2	Cell 147	4	4
Cell 148	2	2	Cell 148	4	4
Cell 149	2	2	Cell 149	4	4
Cell 150	2	2	Cell 150	4	4
Cell 151	2	2	Cell 151	4	4
Cell 152	2	2	Cell 152	4	4
Cell 153	2	2	Cell 153	4	4
Cell 154	2	2	Cell 154	4	4
Cell 155	2	2	Cell 155	4	4
Cell 156	2	2	Cell 156	4	4
Cell 157	2	2	Cell 157	4	4
Cell 158	2	2	Cell 158	4	4
Cell 159	2	2	Cell 159	4	4
Cell 160	2	2	Cell 160	4	4
Cell 161	2	2	Cell 161	4	4
Cell 162	2	2	Cell 162	4	4
Cell 163	2	2	Cell 163	4	4
Cell 164	2	2	Cell 164	4	4
Cell 165	2	2	Cell 165	4	4

Cell 166	2	2	Cell 166	4	4
Cell 167	2	2	Cell 167	4	4
Cell 168	2	2	Cell 168	4	4
Cell 169	2	2	Cell 169	4	4
Cell 170	2	2	Cell 170	4	4
Cell 171	2	2	Cell 171	4	4
Cell 172	2	2	Cell 172	4	4
Cell 173	2	2	Cell 173	4	4
Cell 174	2	2	Cell 174	4	4
Cell 175	2	2	Cell 175	4	4
Cell 176	2	2	Cell 176	4	4
Cell 177	2	2	Cell 177	4	4
Cell 178	2	2	Cell 178	4	4
Cell 179	2	2	Cell 179	4	4
Cell 180	2	2	Cell 180	4	4
Cell 181	2	2	Cell 181	4	4
Cell 182	2	2	Cell 182	4	4
Cell 183	2	2	Cell 183	4	4
Cell 184	2	2	Cell 184	4	4
Cell 185	2	2	Cell 185	4	4
Cell 186	2	2	Cell 186	4	4
Cell 187	2	2	Cell 187	4	4
Cell 188	2	2	Cell 188	4	4
Cell 189	2	2	Cell 189	4	4
Cell 190	2	2	Cell 190	4	4
Cell 191	2	2	Cell 191	4	4
Cell 192	2	2	Cell 192	4	4
Cell 193	2	2	Cell 193	4	4
Cell 194	2	2	Cell 194	4	4
Cell 195	2	2	Cell 195	4	4
Cell 196	2	2	Cell 196	4	4
Cell 197	2	2	Cell 197	4	4
Cell 198	2	2	Cell 198	4	4
Cell 199	2	2	Cell 199	4	4
Cell 200	2	2	Cell 200	4	4
Cell 201	2	2	Cell 201	4	4
Cell 202	2	2	Cell 202	4	4
Cell 203	2	2	Cell 203	4	4
Cell 204	2	2	Cell 204	4	4
Cell 205	2	2	Cell 205	4	4
Cell 206	2	2	Cell 206	4	4
Cell 207	2	2	Cell 207	4	4
Cell 208	2	2	Cell 208	4	4

Cell 209	2	2	Cell 209	4	4
Cell 210	2	2	Cell 210	4	4
Cell 211	2	2	Cell 211	4	4
Cell 212	2	2	Cell 212	4	4
Cell 213	2	2	Cell 213	4	4
Cell 214	2	2	Cell 214	4	4
Cell 215	2	2	Cell 215	4	4
Cell 216	2	2	Cell 216	4	4
Cell 217	2	2	Cell 217	4	4
Cell 218	2	2	Cell 218	4	4
Cell 219	2	2	Cell 219	4	4
Cell 220	2	2	Cell 220	4	4
Cell 221	2	2	Cell 221	4	4
Cell 222	2	2	Cell 222	4	4
Cell 223	2	2	Cell 223	4	4
Cell 224	2	2	Cell 224	4	4
Cell 225	2	2	Cell 225	4	4
Cell 226	2	2	Cell 226	4	4
Cell 227	2	2	Cell 227	4	4
Cell 228	2	2	Cell 228	4	4
Cell 229	2	2	Cell 229	4	4
Cell 230	2	2	Cell 230	4	4
Cell 231	2	2	Cell 231	4	4
Cell 232	2	2	Cell 232	4	4
Cell 233	2	2	Cell 233	4	4
Cell 234	2	2	Cell 234	4	4
Cell 235	2	2	Cell 235	4	4
Cell 236	2	2	Cell 236	4	4
Cell 237	2	2	Cell 237	4	4
Cell 238	2	2	Cell 238	4	4
Cell 239	2	2	Cell 239	4	4
Cell 240	2	2	Cell 240	4	4
Cell 241	2	2	Cell 241	4	4
Cell 242	2	2	Cell 242	4	4
Cell 243	2	2	Cell 243	4	4
Cell 244	2	2	Cell 244	4	4
Cell 245	2	2	Cell 245	4	4
Cell 246	2	2	Cell 246	4	4
Cell 247	2	2	Cell 247	4	4
Cell 248	2	2	Cell 248	4	4
Cell 249	2	2	Cell 249	4	4
Cell 250	2	2	Cell 250	4	4
Cell 251	2	2	Cell 251	4	4

Cell 252	2	2	Cell 252	4	4
Cell 253	2	2	Cell 253	4	4
Cell 254	2	2	Cell 254	4	4
Cell 255	2	2	Cell 255	4	4
Cell 256	2	2	Cell 256	4	4
Cell 257	2	2	Cell 257	4	4
Cell 258	2	2	Cell 258	4	4
Cell 259	2	2	Cell 259	4	4
Cell 260	2	2	Cell 260	4	4
Cell 261	2	2	Cell 261	4	4
Cell 262	2	2	Cell 262	4	4
Cell 263	2	2	Cell 263	4	4
Cell 264	2	2	Cell 264	4	4
Cell 265	2	2	Cell 265	4	4
Cell 266	2	2	Cell 266	4	4
Cell 267	2	2	Cell 267	4	4
Cell 268	2	2	Cell 268	4	4
Cell 269	2	2	Cell 269	4	4
Cell 270	2	2	Cell 270	4	4
Cell 271	2	2	Cell 271	4	4
Cell 272	2	2	Cell 272	4	4
Cell 273	2	2	Cell 273	4	4
Cell 274	2	2	Cell 274	4	4
Cell 275	2	2	Cell 275	4	4
Cell 276	2	2	Cell 276	4	4
Cell 277	2	2	Cell 277	4	4
Cell 278	2	2	Cell 278	4	4
Cell 279	2	2	Cell 279	4	4
Cell 280	2	2	Cell 280	4	4
Cell 281	2	2	Cell 281	4	4
Cell 282	2	2	Cell 282	4	4
Cell 283	2	2	Cell 283	4	4
Cell 284	2	2	Cell 284	4	4
Cell 285	2	2	Cell 285	4	4
Cell 286	2	2	Cell 286	4	4
Cell 287	2	2	Cell 287	4	4
Cell 288	2	2	Cell 288	4	4
Cell 289	2	2	Cell 289	4	4
Cell 290	2	2	Cell 290	4	4
Cell 291	2	2	Cell 291	4	4
Cell 292	2	2	Cell 292	4	4
Cell 293	2	2	Cell 293	4	4
Cell 294	2	2	Cell 294	4	4

Cell 295	2	2	Cell 295	4	4
Cell 296	2	2	Cell 296	4	4
Cell 297	2	2	Cell 297	4	4
Cell 298	2	2	Cell 298	4	4
Cell 299	2	2	Cell 299	4	4
Cell 300	2	2	Cell 300	4	4
Cell 301	2	2	Cell 301	4	4
Cell 302	2	2	Cell 302	4	4
Cell 303	2	2	Cell 303	4	4
Cell 304	2	2	Cell 304	4	4
Cell 305	2	2	Cell 305	4	4
Cell 306	2	2	Cell 306	4	4
Cell 307	2	2	Cell 307	4	4
Cell 308	2	2	Cell 308	4	4
Cell 309	2	2	Cell 309	4	4
Cell 310	2	2	Cell 310	4	4
Cell 311	2	2	Cell 311	4	4
Cell 312	2	2	Cell 312	4	4
Cell 313	2	2	Cell 313	4	4
Cell 314	2	2	Cell 314	4	4
Cell 315	2	2	Cell 315	4	4
Cell 316	2	2	Cell 316	4	4
Cell 317	2	2	Cell 317	4	4
Cell 318	2	2	Cell 318	4	4
Cell 319	2	2	Cell 319	4	4
Cell 320	2	2	Cell 320	4	4
Cell 321	2	2	Cell 321	4	4
Cell 322	2	2	Cell 322	4	4
Cell 323	2	2	Cell 323	4	4
Cell 324	2	2	Cell 324	4	4
Cell 325	2	2	Cell 325	4	4
Cell 326	2	2	Cell 326	4	4
Cell 327	2	2	Cell 327	4	4
Cell 328	2	2	Cell 328	4	4
Cell 329	2	2	Cell 329	4	4
Cell 330	2	2	Cell 330	4	4
Cell 331	2	2	Cell 331	4	4
Cell 332	2	2	Cell 332	4	4
Cell 333	2	2	Cell 333	4	4
Cell 334	2	2	Cell 334	4	4
Cell 335	2	2	Cell 335	4	4
Cell 336	2	2	Cell 336	4	4
Cell 337	2	2	Cell 337	4	4

Cell 338	2	2	Cell 338	4	4
Cell 339	2	2	Cell 339	4	4
Cell 340	2	2	Cell 340	4	4
Cell 341	2	2	Cell 341	4	4
Cell 342	2	2	Cell 342	4	4
Cell 343	2	2	Cell 343	4	4
Cell 344	2	2	Cell 344	4	4
Cell 345	2	2	Cell 345	4	4
Cell 346	2	2	Cell 346	4	4
Cell 347	2	2	Cell 347	4	4
Cell 348	2	2	Cell 348	4	4
Cell 349	2	2	Cell 349	4	4
Cell 350	2	2	Cell 350	4	4
Cell 351	2	2	Cell 351	4	4
Cell 352	2	2	Cell 352	4	4
Cell 353	2	2	Cell 353	**4**	**4**
Cell 354	2	2	Cell 354	4	3
Cell 355	2	2	Cell 355	4	3
Cell 356	2	2	Cell 356	4	3
Cell 357	2	2	Cell 357	4	3
Cell 358	2	2	Cell 358	4	3
Cell 359	2	2	Cell 359	4	3
Cell 360	2	2	Cell 360	4	3
Cell 361	2	2	Cell 361	4	3
Cell 362	2	2	Cell 362	4	3
Cell 363	2	2	Cell 363	4	2
Cell 364	2	2	Cell 364	4	2
Cell 365	2	2	Cell 365	4	2
Cell 366	2	2	Cell 366	4	2
Cell 367	2	2	Cell 367	4	2
Cell 368	2	2	Cell 368	4	2
Cell 369	2	2	Cell 369	4	2
Cell 370	2	2	Cell 370	4	2
Cell 371	2	2	Cell 371	3	7
Cell 372	2	2	Cell 372	3	5
Cell 373	2	2	Cell 373	3	4
Cell 374	2	2	Cell 374	3	4
Cell 375	2	2	Cell 375	3	4
Cell 376	2	2	Cell 376	3	4
Cell 377	2	2	Cell 377	3	4
Cell 378	2	2	Cell 378	3	4
Cell 379	2	2	Cell 379	3	4
Cell 380	2	2	Cell 380	3	4

Cell 381	2	2	Cell 381	3	4
Cell 382	2	2	Cell 382	3	4
Cell 383	2	2	Cell 383	3	4
Cell 384	2	2	Cell 384	3	3
Cell 385	2	2	Cell 385	3	3
Cell 386	2	2	Cell 386	3	3
Cell 387	2	2	Cell 387	3	3
Cell 388	2	2	Cell 388	3	3
Cell 389	2	2	Cell 389	3	3
Cell 390	2	2	Cell 390	3	3
Cell 391	2	2	Cell 391	3	3
Cell 392	2	2	Cell 392	3	2
Cell 393	2	2	Cell 393	3	2
Cell 394	2	2	Cell 394	3	2
Cell 395	2	2	Cell 395	3	2
Cell 396	2	2	Cell 396	3	2
Cell 397	2	2	Cell 397	3	2
Cell 398	2	2	Cell 398	3	2
Cell 399	2	2	Cell 399	3	2
Cell 400	2	2	Cell 400	3	2
Cell 401	2	2	Cell 401	3	2
Cell 402	2	2	Cell 402	3	2
Cell 403	2	2	Cell 403	3	2
Cell 404	2	2	Cell 404	3	2
Cell 405	2	2	Cell 405	3	2
Cell 406	2	2	Cell 406	2	5
Cell 407	2	2	Cell 407	2	5
Cell 408	2	2	Cell 408	2	4
Cell 409	2	2	Cell 409	2	4
Cell 410	2	2	Cell 410	2	4
Cell 411	2	2	Cell 411	2	4
Cell 412	2	2	Cell 412	2	4
Cell 413	2	2	Cell 413	2	4
Cell 414	2	2	Cell 414	2	4
Cell 415	2	2	Cell 415	2	4
Cell 416	2	2	Cell 416	2	4
Cell 417	2	2	Cell 417	2	4
Cell 418	2	2	Cell 418	2	4
Cell 419	2	2	Cell 419	2	4
Cell 420	2	2	Cell 420	2	4
Cell 421	2	2	Cell 421	2	4
Cell 422	2	2	Cell 422	2	4
Cell 423	2	2	Cell 423	2	4

Cell 424	2	2	Cell 424	2	4
Cell 425	2	2	Cell 425	2	4
Cell 426	2	2	Cell 426	2	4
Cell 427	2	2	Cell 427	2	3
Cell 428	2	2	Cell 428	2	3
Cell 429	2	2	Cell 429	2	3
Cell 430	2	2	Cell 430	2	3
Cell 431	2	2	Cell 431	2	3
Cell 432	2	2	Cell 432	2	3
Cell 433	2	2	Cell 433	2	3
Cell 434	2	2	Cell 434	2	3
Cell 435	2	2	Cell 435	2	3
Cell 436	2	2	Cell 436	2	3
Cell 437	2	2	Cell 437	2	3
Cell 438	2	2	Cell 438	2	3
Cell 439	2	2	Cell 439	2	3
Cell 440	2	2	Cell 440	2	3
Cell 441	2	2	Cell 441	2	3
Cell 442	2	2	Cell 442	2	3
Cell 443	2	2	Cell 443	2	3
Cell 444	2	2	Cell 444	2	3
Cell 445	2	2	Cell 445	2	3
Cell 446	2	2	Cell 446	2	3
Cell 447	2	2	Cell 447	2	3
Cell 448	2	2	Cell 448	2	3
Cell 449	2	2	Cell 449	2	3
Cell 450	2	2	Cell 450	2	2
Cell 451	2	2	Cell 451	2	2
Cell 452	2	2	Cell 452	2	2
Cell 453	2	2	Cell 453	2	2
Cell 454	2	2	Cell 454	2	2
Cell 455	2	2	Cell 455	2	2
Cell 456	2	2	Cell 456	2	2
Cell 457	2	2	Cell 457	2	2
Cell 458	2	2	Cell 458	2	2
Cell 459	2	2	Cell 459	2	2
Cell 460	2	2	Cell 460	2	2
Cell 461	2	2	Cell 461	2	2
Cell 462	2	2	Cell 462	2	2
Cell 463	2	2	Cell 463	2	2
Cell 464	2	2	Cell 464	2	2
Cell 465	2	2	Cell 465	2	2
Cell 466	2	2	Cell 466	2	2

Cell 467	2	2	Cell 467	2	2
Cell 468	2	2	Cell 468	2	2
Cell 469	2	2	Cell 469	2	2
Cell 470	2	2	Cell 470	2	2
Cell 471	2	2	Cell 471	2	2
Cell 472	2	2	Cell 472	2	2
Cell 473	2	2	Cell 473	2	2
Cell 474	2	2	Cell 474	2	2
Cell 475	2	2	Cell 475	2	2
Cell 476	2	2	Cell 476	2	2
Cell 477	2	2	Cell 477	2	2
Cell 478	2	2	Cell 478	2	2
Cell 479	2	2	Cell 479	2	2
Cell 480	2	2	Cell 480	2	2
Cell 481	2	2	Cell 481	2	2
Cell 482	2	2	Cell 482	2	2
Cell 483	2	2	Cell 483	2	2
Cell 484	2	2	Cell 484	2	2
Cell 485	2	2	Cell 485	2	2
Cell 486	2	2	Cell 486	2	2
Cell 487	2	2	Cell 487	2	2
Cell 488	2	2	Cell 488	2	2
Cell 489	2	2	Cell 489	2	2
Cell 490	2	2	Cell 490	2	2
Cell 491	2	2	Cell 491	2	2
Cell 492	2	2	Cell 492	2	2
Cell 493	2	2	Cell 493	2	2
Cell 494	2	2	Cell 494	2	2
Cell 495	2	2	Cell 495	2	2
Cell 496	2	2	Cell 496	2	2
Cell 497	2	2	Cell 497	2	2
Cell 498	2	2	Cell 498	2	2
Cell 499	2	2	Cell 499	2	2
Cell 500	2	2	Cell 500	2	2
Cell 501	2	2	Cell 501	2	2
Cell 502	2	2	Cell 502	2	2
Cell 503	2	2	Cell 503	2	2
Cell 504	2	2	Cell 504	2	2
Cell 505	2	2	Cell 505	2	2
Cell 506	2	2	Cell 506	2	2
Cell 507	2	2	Cell 507	2	2
Cell 508	2	2	Cell 508	2	2
Cell 509	2	2	Cell 509	2	2

Cell 510	2	2	Cell 510	2	2
Cell 511	2	2	Cell 511	2	2
Cell 512	2	2	Cell 512	2	2
Cell 513	2	2	Cell 513	2	2
Cell 514	2	2	Cell 514	2	2
Cell 515	2	2	Cell 515	2	2
Cell 516	2	2	Cell 516	2	2
Cell 517	2	2	Cell 517	2	2
Cell 518	2	2	Cell 518	2	2
Cell 519	2	2	Cell 519	2	2
Cell 520	2	2	Cell 520	2	2
Cell 521	2	2	Cell 521	2	2
Cell 522	2	2	Cell 522	2	2
Cell 523	2	2	Cell 523	2	2
Cell 524	2	2	Cell 524	2	2
Cell 525	2	2	Cell 525	2	2
Cell 526	2	2	Cell 526	2	2
Cell 527	2	2	Cell 527	2	2
Cell 528	2	2	Cell 528	2	2
Cell 529	2	2	Cell 529	2	2
Cell 530	2	2	Cell 530	2	2
Cell 531	2	2	Cell 531	2	2
Cell 532	2	2	Cell 532	2	2
Cell 533	2	2	Cell 533	2	2
Cell 534	2	2	Cell 534	2	2
Cell 535	2	2	Cell 535	2	2
Cell 536	2	2	Cell 536	2	2
Cell 537	2	2	Cell 537	2	2
Cell 538	2	2	Cell 538	2	2
Cell 539	2	2	Cell 539	2	2
Cell 540	2	2	Cell 540	2	2
Cell 541	2	2	Cell 541	2	2
Cell 542	2	2	Cell 542	2	2
Cell 543	2	2	Cell 543	2	2
Cell 544	2	2	Cell 544	2	2
Cell 545	2	2	Cell 545	2	2
Cell 546	2	2	Cell 546	2	2
Cell 547	2	2	Cell 547	2	2
Cell 548	2	2	Cell 548	2	2
Cell 549	2	2	Cell 549	2	2
Cell 550	2	2	Cell 550	2	2
Cell 551	2	2	Cell 551	2	2
Cell 552	2	2	Cell 552	2	2

Cell 553	2	2	Cell 553	2	2
Cell 554	2	2	Cell 554	2	2
Cell 555	2	2	Cell 555	2	2
Cell 556	2	2	Cell 556	2	2
Cell 557	2	2	Cell 557	2	2
Cell 558	2	2	Cell 558	2	2
Cell 559	2	2	Cell 559	2	2
Cell 560	2	2	Cell 560	2	2
Cell 561	2	2	Cell 561	2	2
Cell 562	2	2	Cell 562	2	2
Cell 563	2	2	Cell 563	2	2
Cell 564	2	2	Cell 564	2	2
Cell 565	2	2	Cell 565	2	2
Cell 566	2	2	Cell 566	2	2
Cell 567	2	2	Cell 567	2	2
Cell 568	2	2	Cell 568	2	2
Cell 569	2	2	Cell 569	2	2
Cell 570	2	2	Cell 570	2	2
Cell 571	2	2	Cell 571	2	2
Cell 572	2	2	Cell 572	2	2
Cell 573	2	2	Cell 573	2	2
Cell 574	2	2	Cell 574	2	2
Cell 575	2	2	Cell 575	2	2
Cell 576	2	2	Cell 576	2	2
Cell 577	2	2	Cell 577	2	2
Cell 578	2	2	Cell 578	2	2
Cell 579	2	2	Cell 579	2	2
Cell 580	2	2	Cell 580	2	2
Cell 581	2	2	Cell 581	2	2
Cell 582	2	2	Cell 582	2	2
Cell 583	2	2	Cell 583	2	2
Cell 584	2	2	Cell 584	2	2
Cell 585	2	2	Cell 585	2	2
Cell 586	2	2	Cell 586	2	2
Cell 587	2	2	Cell 587	2	2
Cell 588	2	2	Cell 588	2	2
Cell 589	2	2	Cell 589	2	2
Cell 590	2	2	Cell 590	2	2
Cell 591	2	2	Cell 591	2	2
Cell 592	2	2	Cell 592	2	2
Cell 593	2	2	Cell 593	2	2
Cell 594	2	2	Cell 594	2	2
Cell 595	2	2	Cell 595	2	2

Cell 596	2	2
Cell 597	2	2
Cell 598	2	2
Cell 599	2	2
Cell 600	2	2
Cell 601	2	2
Cell 602	2	2
Cell 603	2	2
Cell 604	2	2
Cell 605	2	2

Cell 596	2	2
Cell 597	2	2
Cell 598	2	2
Cell 599	2	2
Cell 600	2	2
Cell 601	2	2
Cell 602	2	2
Cell 603	2	2
Cell 604	2	2
Cell 605	2	2
Cell 606	2	2
Cell 607	2	2
Cell 608	2	2
Cell 609	2	2
Cell 610	2	2
Cell 611	2	2
Cell 612	2	2
Cell 613	2	2
Cell 614	2	2
Cell 615	2	2
Cell 616	2	2
Cell 617	2	2
Cell 618	2	2
Cell 619	2	2
Cell 620	2	2
Cell 621	2	2
Cell 622	2	2
Cell 623	2	2
Cell 624	2	2
Cell 625	2	2
Cell 626	2	2
Cell 627	2	2
Cell 628	2	2
Cell 629	2	2
Cell 630	2	2
Cell 631	2	2
Cell 632	2	2
Cell 633	2	2
Cell 634	2	2
Cell 635	2	2
Cell 636	2	2
Cell 637	2	2
Cell 638	2	2

Cell 639	2	2
Cell 640	2	2
Cell 641	2	2
Cell 642	2	2
Cell 643	2	2
Cell 644	2	2
Cell 645	2	2
Cell 646	2	2
Cell 647	2	2
Cell 648	2	2
Cell 649	2	2
Cell 650	2	2
Cell 651	2	2
Cell 652	2	2
Cell 653	2	2
Cell 654	2	2
Cell 655	2	2
Cell 656	2	2
Cell 657	2	2
Cell 658	2	2
Cell 659	2	2
Cell 660	2	2
Cell 661	2	2
Cell 662	2	2
Cell 663	2	2
Cell 664	2	2
Cell 665	2	2
Cell 666	2	2
Cell 667	2	2
Cell 668	2	2
Cell 669	2	2
Cell 670	2	2
Cell 671	2	2
Cell 672	2	2
Cell 673	2	2
Cell 674	2	2
Cell 675	2	2
Cell 676	2	2
Cell 677	2	2
Cell 678	2	2
Cell 679	2	2
Cell 680	2	2
Cell 681	2	2

Cell 682	2	2
Cell 683	2	2
Cell 684	2	2
Cell 685	2	2
Cell 686	2	2
Cell 687	2	2
Cell 688	2	2
Cell 689	2	2
Cell 690	2	2
Cell 691	2	2
Cell 692	2	2
Cell 693	2	2
Cell 694	2	2
Cell 695	2	2
Cell 696	2	2
Cell 697	2	2
Cell 698	2	2
Cell 699	2	2
Cell 700	2	2
Cell 701	2	2
Cell 702	2	2
Cell 703	2	2
Cell 704	2	2
Cell 705	2	2
Cell 706	2	2
Cell 707	2	2
Cell 708	2	2
Cell 709	2	2
Cell 710	2	2
Cell 711	2	2
Cell 712	2	2
Cell 713	2	2
Cell 714	2	2
Cell 715	2	2
Cell 716	2	2
Cell 717	2	2
Cell 718	2	2
Cell 719	2	2
Cell 720	2	2
Cell 721	2	2
Cell 722	2	2
Cell 723	2	2
Cell 724	2	2

Cell 725	2	2
Cell 726	2	2
Cell 727	2	2
Cell 728	2	2
Cell 729	2	2
Cell 730	2	2
Cell 731	2	2
Cell 732	2	2
Cell 733	2	2
Cell 734	2	2
Cell 735	2	2
Cell 736	2	2
Cell 737	2	2
Cell 738	2	2
Cell 739	2	2
Cell 740	2	2
Cell 741	2	2
Cell 742	2	2
Cell 743	2	2
Cell 744	2	2
Cell 745	2	2
Cell 746	2	2
Cell 747	2	2
Cell 748	2	2
Cell 749	2	2
Cell 750	2	2
Cell 751	2	2
Cell 752	2	2
Cell 753	2	2
Cell 754	2	2
Cell 755	2	2
Cell 756	2	2
Cell 757	2	2
Cell 758	2	2
Cell 759	2	2
Cell 760	2	2
Cell 761	2	2
Cell 762	2	2
Cell 763	2	2
Cell 764	2	2
Cell 765	2	2
Cell 766	2	2
Cell 767	2	2

Cell 768	2	2
Cell 769	2	2
Cell 770	2	2
Cell 771	2	2
Cell 772	2	2
Cell 773	2	2
Cell 774	2	2
Cell 775	2	2
Cell 776	2	2
Cell 777	2	2
Cell 778	2	2
Cell 779	2	2
Cell 780	2	2
Cell 781	2	2
Cell 782	2	2
Cell 783	2	2
Cell 784	2	2
Cell 785	2	2
Cell 786	2	2
Cell 787	2	2
Cell 788	2	2
Cell 789	2	2
Cell 790	2	2
Cell 791	2	2
Cell 792	2	2
Cell 793	2	2
Cell 794	2	2
Cell 795	2	2
Cell 796	2	2
Cell 797	2	2
Cell 798	2	2
Cell 799	2	2
Cell 800	2	2
Cell 801	2	2
Cell 802	2	2
Cell 803	2	2
Cell 804	2	2
Cell 805	2	2
Cell 806	2	2
Cell 807	2	2
Cell 808	2	2
Cell 809	2	2
Cell 810	2	2

Cell 811	2	2
Cell 812	2	2
Cell 813	2	2
Cell 814	2	2
Cell 815	2	2
Cell 816	2	2
Cell 817	2	2
Cell 818	2	2
Cell 819	2	2
Cell 820	2	2
Cell 821	2	2
Cell 822	2	2
Cell 823	2	2
Cell 824	2	2
Cell 825	2	2
Cell 826	2	2
Cell 827	2	2
Cell 828	2	2
Cell 829	2	2
Cell 830	2	2
Cell 831	2	2
Cell 832	2	2
Cell 833	2	2
Cell 834	2	2
Cell 835	2	2
Cell 836	2	2
Cell 837	2	2
Cell 838	2	2
Cell 839	2	2
Cell 840	2	2
Cell 841	2	2
Cell 842	2	2
Cell 843	2	2
Cell 844	2	2
Cell 845	2	2
Cell 846	2	2
Cell 847	2	2
Cell 848	2	2
Cell 849	2	2
Cell 850	2	2
Cell 851	2	2
Cell 852	2	2
Cell 853	2	2

Cell 854	2	2
Cell 855	2	2
Cell 856	2	2
Cell 857	2	2
Cell 858	2	2
Cell 859	2	2
Cell 860	2	2
Cell 861	2	2
Cell 862	2	2
Cell 863	2	2
Cell 864	2	2
Cell 865	2	2
Cell 866	2	2
Cell 867	2	2
Cell 868	2	2
Cell 869	2	2
Cell 870	2	2
Cell 871	2	2
Cell 872	2	2
Cell 873	2	2
Cell 874	2	2
Cell 875	2	2
Cell 876	2	2
Cell 877	2	2
Cell 878	2	2
Cell 879	2	2
Cell 880	2	2
Cell 881	2	2
Cell 882	2	2
Cell 883	2	2
Cell 884	2	2
Cell 885	2	2
Cell 886	2	2
Cell 887	2	2
Cell 888	2	2
Cell 889	2	2
Cell 890	2	2
Cell 891	2	2
Cell 892	2	2
Cell 893	2	2
Cell 894	2	2
Cell 895	2	2
Cell 896	2	2

Cell 897	2	2
Cell 898	2	2
Cell 899	2	1
Cell 900	2	1
Cell 901	2	1
Cell 902	1	3
Cell 903	1	2
Cell 904	1	2
Cell 905	1	1
